# Supplementary material for: Small molecules enhance the potency of natural antimicrobial peptides
Source: Biophys J. 2021 Dec 24;121(3):491–501. doi: 10.1016/j.bpj.2021.12.029 (PMC8822605; doi:10.1016/j.bpj.2021.12.029)
Supplement: Document S1. Figures S1–S8 and Tables S1 and S2 [file mmc1.pdf]

Biophysical Journal, Volume *121*

Supplemental information

Small molecules enhance the potency of natural antimicrobial peptides

Valeria Losasso, Khushbu Agarwal, Morris Waskar, Amitabha Majumdar, Jason Crain, Martyn Winn, and Michael Hoptroff

Supporting information

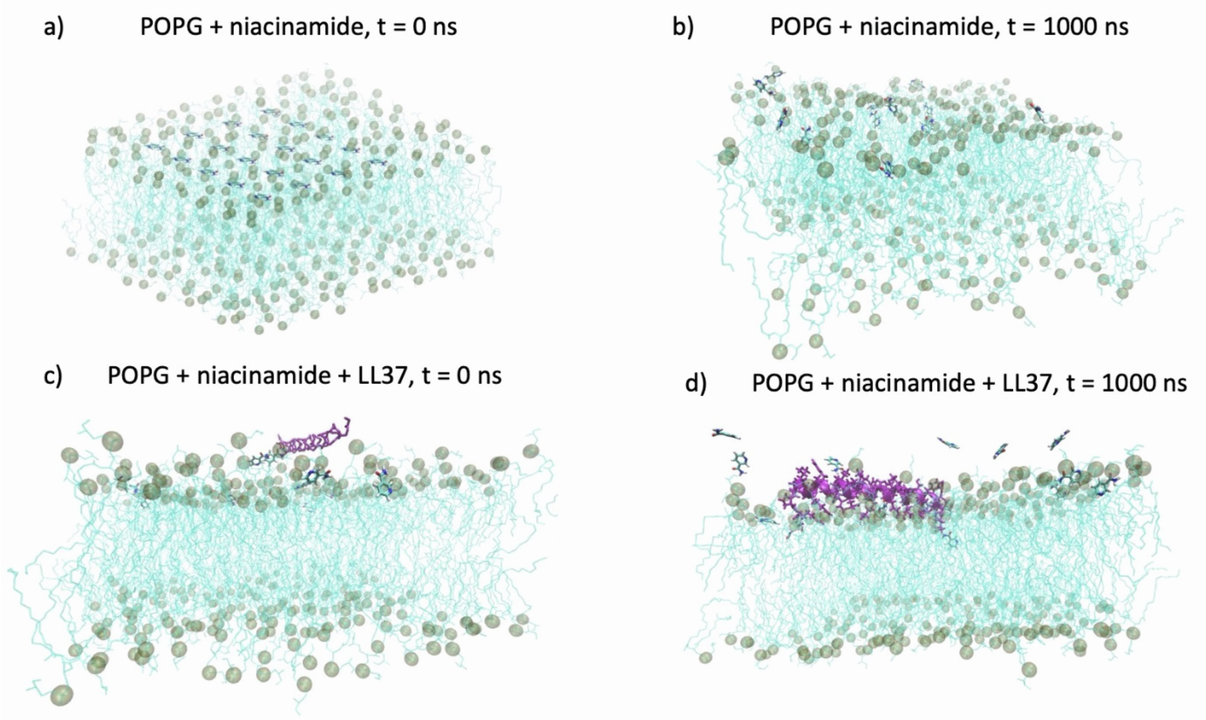

Fig. S1. a) Simulation setup for a membrane system (POPG as an example) with 20 additives in a grid (niacinamide as an example). b) Snapshot of the first POPG + niacinamide simulation at t = 1000 ns. c) Addition of LL-37 peptide onto the system. d) Snapshot of the first POPG + LL-37 + niacinamide simulation at t = 1000 ns.

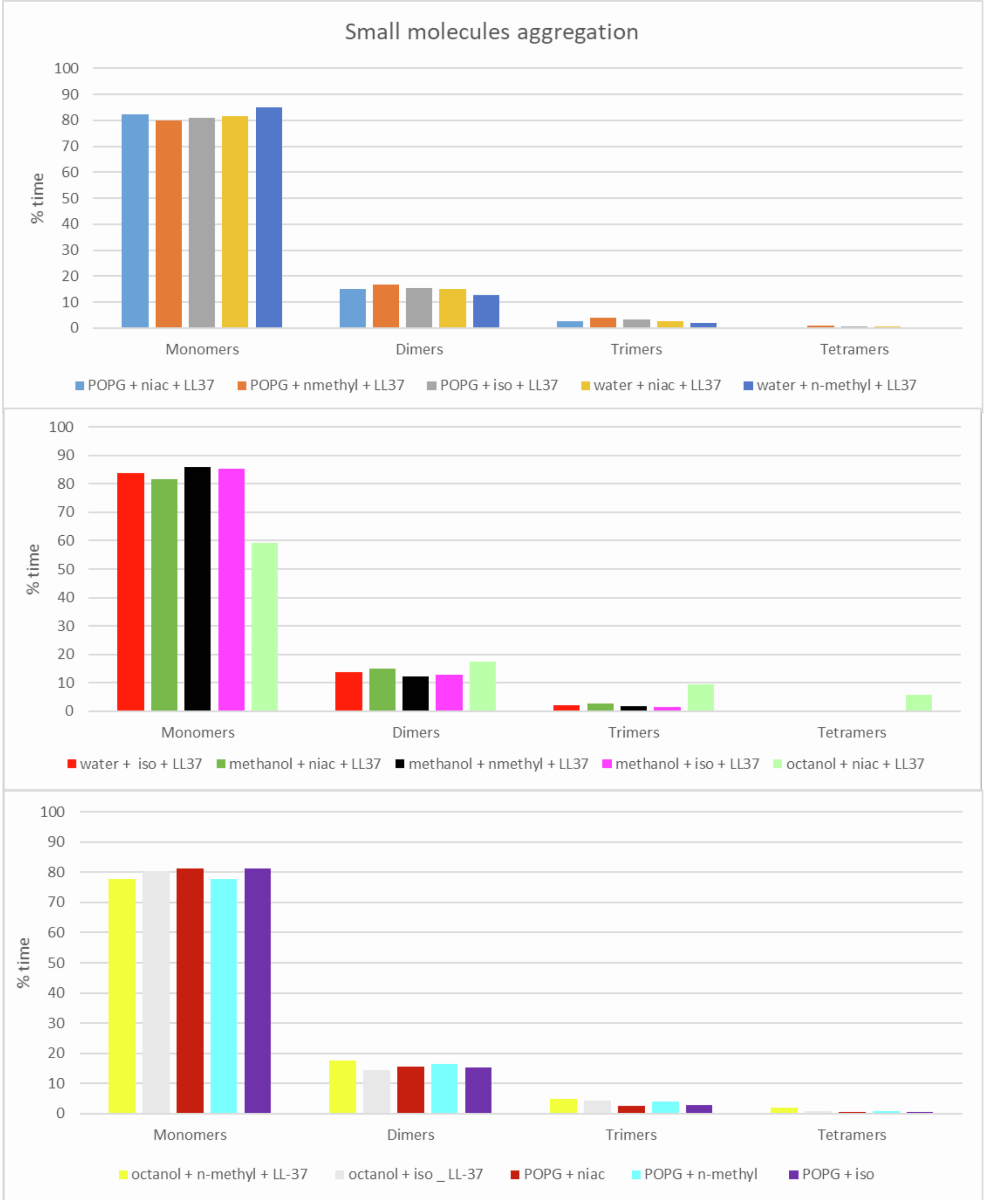

Fig. S2. Percentage over simulation time of aggregates in different sizes for the three additives in membrane, water, methanol and octanol. In every group, at least 1 molecule is located < 5 Å away from any of the others.

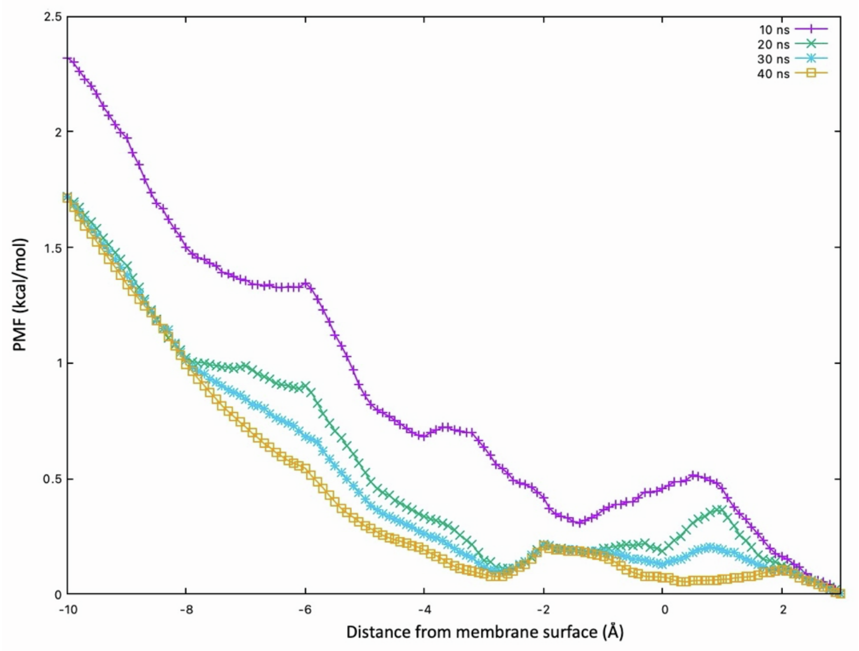

Fig. S3. PMF profile calculated after 10, 20, 30 and 40 ns for POPG + niacinamide.

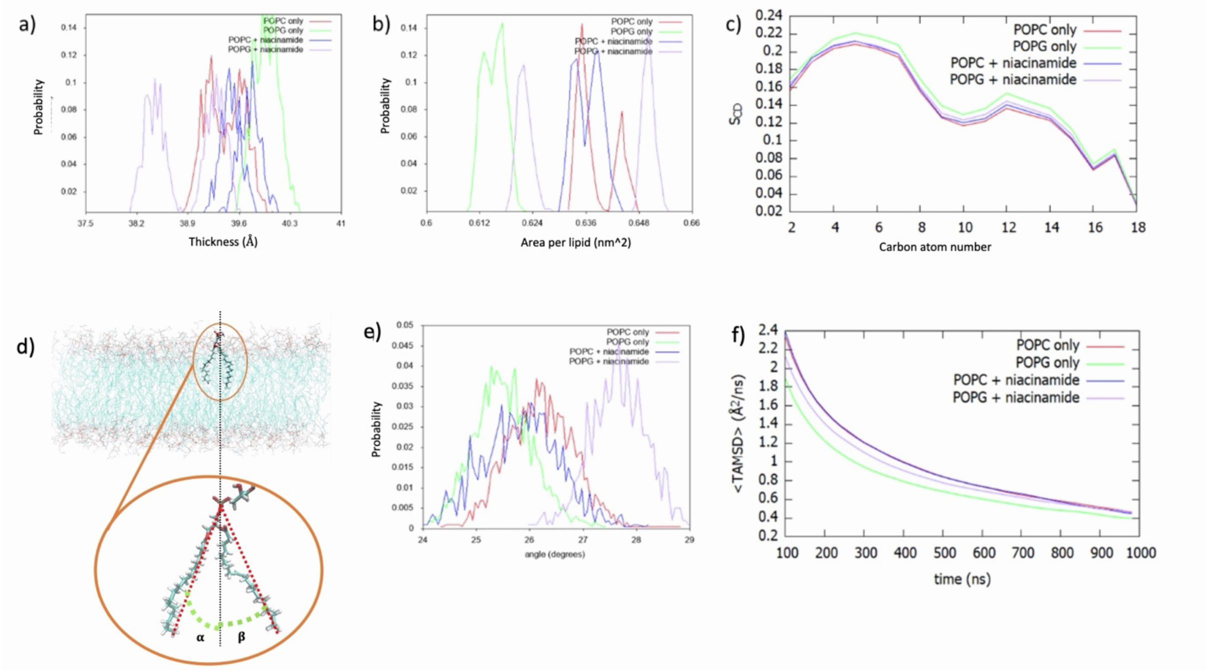

Fig. S4. Analysis of unbiased simulations for niacinamide in complex with POPC or POPG membranes. a) Distribution of thickness values; b) Distribution of area per lipid values; c) S_CD order parameter; d) Definition of tilt angle as the average of α and β angles, formed between the membrane normal at t = 0 and the line connecting the lipid phosphate to the terminal carbon atom of each tail; e) Distribution of lipid tilt angles; f) Ensemble averaged time-averaged mean square displacement (TAMSD).

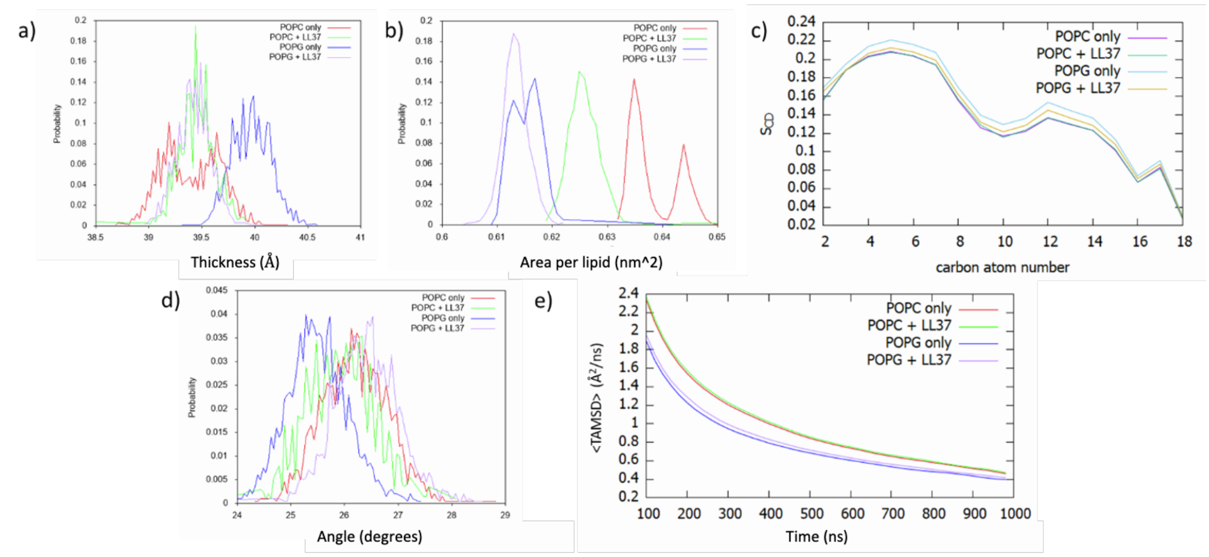

Fig. S5. Analysis of LL-37 in complex with POPC and POPG membranes compared with membrane-only systems. a) Distribution of thickness values; b) Distribution of area per lipid values; c) S_CD order parameter; d) Distribution of lipid tilt angles; e) Ensemble averaged time-averaged mean square displacement (TAMSD).

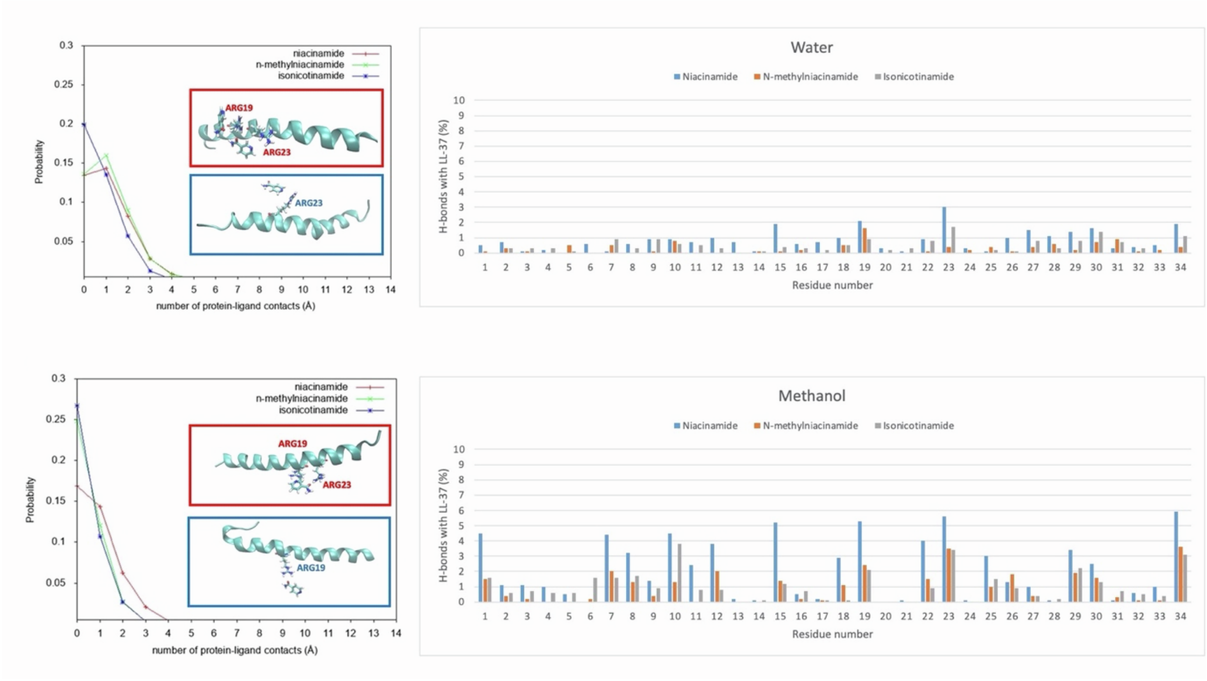

Fig. S6. Distribution of contacts for niacinamide and its derivatives with LL-37 in membrane (top) and octanol (bottom). Representative snapshots for niacinamide and isonicotinamide binding are shown in red and blue boxes, respectively.

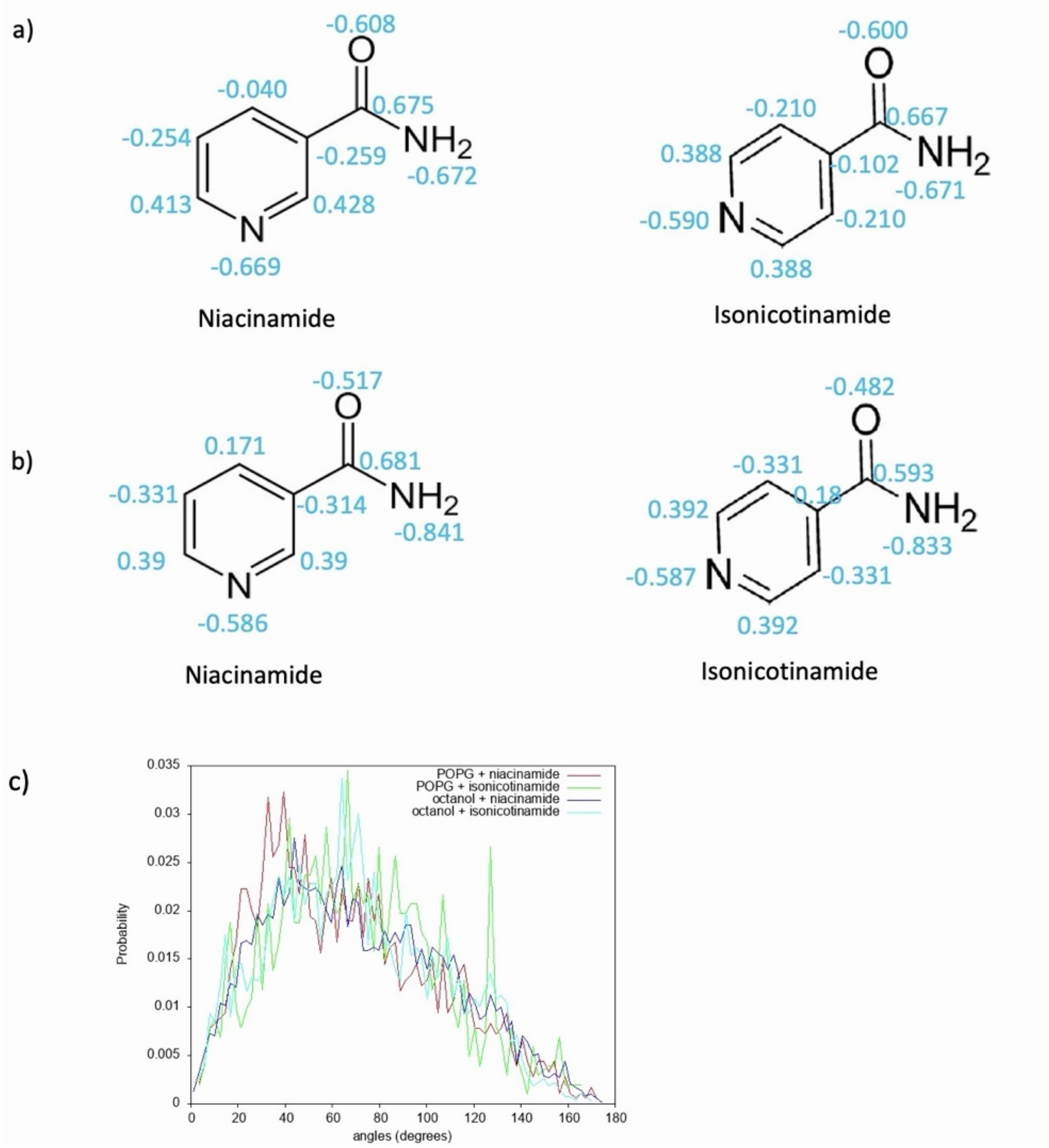

Fig. S7. a) Different charge distribution between niacinamide and isonicotinamide as calculated by Antechamber. b) Partial charges on the two molecules as calculated by restrained electrostatic potential (ESP)-based charge fitting. c) Distribution of angles between LL-37 main axis and niacinamide/isonicotinamide main axis when the two molecules are less than 5 Å apart.

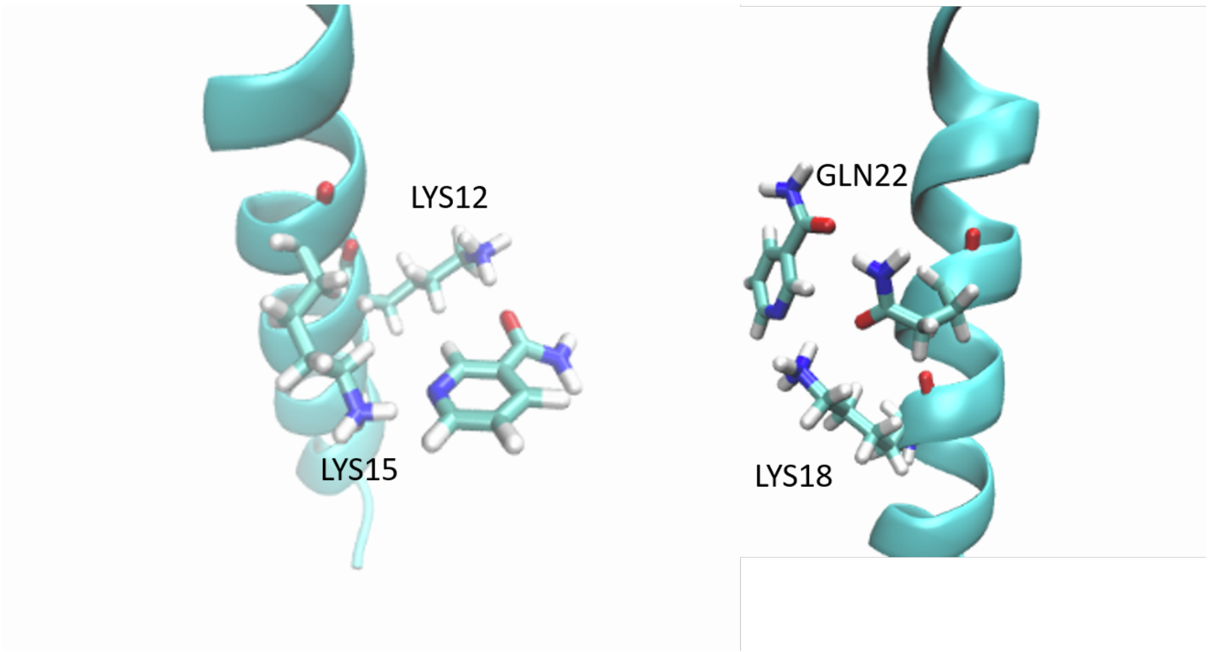

Fig. S8. Simultaneous hydrogen bonds between niacinamide and LL-37 residue pairs.

Table S1. Antimicrobial activity of niacinamide and analogues with LL37 on *S.aureus*

**SET 1**	**Log counts (cfu/mL)**		
	**R1**	**R2**	**Average**
Culture Control	1130000	1220000	1175000
3% NIA	1120000	1590000	1355000
3% MeNAM	1270000	1440000	1355000
3% ISON	1350000	1090000	1220000
LL37 (2 μg/mL)	80800	67200	74000
3% NIA + LL37(2 μg/mL)	6100	5100	5600
3% MeNAM + LL37(2 μg/mL)	14800	11300	13050
3% ISON + LL37(2 μg/mL)	57600	41200	49400

**SET 2**	**Log counts (cfu/mL)**		
	**R1**	**R2**	**Average**
Culture Control	2480000	2320000	2400000
3% NIA	2400000	2480000	2440000
3% MeNAM	2560000	2480000	2520000
3% ISON	2160000	2400000	2280000
LL37 (2 μg/mL)	22400	19600	21000
3% NIA + LL37(2 μg/mL)	1300	1000	1150
3% MeNAM + LL37(2 μg/mL)	2300	1500	1900
3% ISON + LL37(2 μg/mL)	4500	5300	4900

Key: NIA= Niacinamide, MeNAM= N-methylnicotinamide, ISON= Isonicotinamide,
LL37=Cathelicidin Antimicrobial peptide (AMP)
All % values as w/v
R1 and R2 are technical replicates

Table S2. Hydrogen bonds between niacinamide, n-methylnicotinamide and nicotinamide and LL-37 in four different environments (POPG membrane, water, methanol, octanol).

	Ring N	Amide N	Amide O
POPG + niacinamide	Leu2 0.3%<br>Phe5 0.2%<br>Ser9 0.6%<br>Arg7 0.4%<br>Lys8 0.2%<br>Lys10 0.3%<br>Lys15 0.2%<br>Lys18 2.2%<br>Lys25 0.6%<br>Arg23 0.6%<br>Gln22 1%<br>Arg29 0.4%<br>Asn30 0.4%<br>Arg34 0.3%	Leu1 0.4%<br>Leu2 0.7%<br>Gly3 0.2%<br>Asp4 0.4%<br>Phe6 0.2%<br>Arg7 0.5%<br>Ser9 0.4%<br>Lys10 0.2%<br>Glu11 0.6%<br>Lys12 0.1%<br>Ile13 0.2%<br>Gly14 0.2%<br>Lys15 0.3%<br>Glu16 0.3%<br>Lys18 0.3%<br>Arg19 0.6%<br>Ile20 0.3%<br>Val21 0.1%<br>Gln22 2.6%<br>Arg23 0.7%<br>Ile24 0.1%<br>Lys25 0.3%<br>Asp26 1%<br>Phe27 0.2%<br>Leu28 0.2%<br>Arg29 0.4%<br>Asn30 0.6%<br>Leu31 0.3%<br>Val32 0.2%<br>Pro33 0.7%<br>Arg34 0.4%	Leu1 0.9%<br>Leu2 0.3%<br>Gly3 0.9%<br>Asp4 0.1%<br>Phe6 0.1%<br>Arg7 0.4%<br>Lys8 0.4%<br>Ser9 0.1%<br>Lys10 1%<br>Glu11 0.3%<br>Lys12 0.3%<br>Lys15 0.5%<br>Glu16 0.1%<br>Lys18 0.8%<br>Arg19 1%<br>Val21 0.2%<br>Gln22 2.1%<br>Arg23 0.5%<br>Ile24 0.1%<br>Lys25 0.9%<br>Asp26 0.2%<br>Phe27 0.1%<br>Arg29 0.6%<br>Asn30 0.7%<br>Arg34 0.6%
POPG + n-methylnicotinamide	Leu1 0.1%<br>Leu2 0.1%<br>Gly3 0.1%<br>Arg7 0.1%<br>Lys8 0.2%<br>Ser9 0.5%<br>Lys10 0.3%<br>Lys12 0.3%<br>Lys18 0.1%<br>Arg19 1.2%<br>Arg23 0.5%<br>Ile24 0.1%	Leu1 0.3%<br>Leu2 0.2%<br>Phe5 0.4%<br>Lys8 0.1%<br>Ser9 1.2%<br>Lys10 0.2%<br>Glu11 0.2%<br>Lys12 1%<br>Ile13 1%<br>Glu16 0.9%<br>Phe17 1%<br>Gln22 0.1%	Leu1 0.2%<br>Leu2 0.3%<br>Gly3 0.2%<br>Phe5 0.1%<br>Phe6 0.1%<br>Lys8 0.1%<br>Ser9 1.4%<br>Lys10 0.9%<br>Lys12 0.9%<br>Ile13 0.3%<br>Lys15 0.1%<br>Lys18 0.3%

	Lys25 0.3%<br>Arg29 0.1%<br>Asn30 0.9%<br>Arg34 0.4%	Arg23 1.1%<br>Asp26 0.6%<br>Phe27 0.3%<br>Arg29 0.2%<br>Asn30 1.9%<br>Leu31 0.7%<br>Pro33 0.2%<br>Arg34 0.1%	Arg19 1%<br>Ile20 0.3%<br>Val21 0.1%<br>Gln22 0.5%<br>Arg23 2.6%<br>Ile24 0.3%<br>Lys25 3.8%<br>Phe27 0.2%<br>Leu28 0.4%<br>Arg29 1.4%<br>Asn30 1.1%<br>Leu31 0.2%<br>Val32 0.1%<br>Arg34 2%
POPG + isonicotinamide	Arg7 0.1%<br>Ser9 0.2%<br>Lys10 0.1%<br>Lys12 0.1%<br>Lys15 0.1%<br>Arg19 0.2%<br>Gln22 0.2%<br>Arg23 0.1%<br>Lys25 0.6%<br>Arg29 0.2%<br>Asn30 0.3%<br>Arg34 1.3%	Leu1 0.1%<br>Gly3 0.1%<br>Phe5 0.1%<br>Phe6 0.1%<br>Arg7 0.3%<br>Ser9 0.4%<br>Lys10 0.2%<br>Glu11 0.2%<br>Lys12 0.5%<br>Ile13 0.1%<br>Lys15 0.1%<br>Glu16 0.5%<br>Phe17 0.1%<br>Lys18 1.1%<br>Arg19 0.1%<br>Ile20 0.1%<br>Gln22 0.7%<br>Arg23 1.3%<br>Ile24 0.1%<br>Lys25 0.9%<br>Asp26 0.8%<br>Phe27 0.8%<br>Leu28 0.4%<br>Arg29 0.6%<br>Asn30 0.9%<br>Leu31 0.7%<br>Leu32 0.6%<br>Pro33 0.2%<br>Arg34 1.4%	Gly3 0.3%<br>Phe6 0.2%<br>Arg7 0.3%<br>Lys8 0.1%<br>Ser9 0.1%<br>Lys10 0.5%<br>Lys12 0.5%<br>Phe17 0.3%<br>Lys18 1%<br>Gly14 0.1%<br>Arg19 0.1%<br>Ile20 0.2%<br>Gln22 0.1%<br>Arg23 1.4%<br>Lys25 1.3%<br>Asp26 0.1%<br>Arg29 0.2%<br>Arg30 0.9%<br>Pro33 0.1%<br>Arg34 1.4%
niacinamide + water	Leu1 0.1%<br>Leu2 0.1%<br>Gly3 0.1%<br>Phe5 0.1%<br>Arg7 0.1%<br>Lys8 0.3%<br>Ser9 0.2%<br>Lys10 0.1%	Leu1 0.2 %<br>Leu2 0.3%<br>Asp4 0.2%<br>Phe5 0.5%<br>Phe6 0.6%<br>Ser9 0.2%<br>Lys10 0.4%<br>Glu11 0.7%	Leu1 0.2%<br>Leu2 0.3%<br>Phe6 0.2%<br>Arg7 0.9%<br>Lys8 0.3%<br>Ser9 0.5%<br>Lys10 0.4%<br>Lys12 0.6%

	Lys12 0.3%<br>Lys15 0.1%<br>Phe17 0.2%<br>Lys18 0.2%<br>Arg19 0.3%<br>Ile20 0.1%<br>Gln22 0.2%<br>Arg23 0.5%<br>Ile24 0.1%<br>Lys25 0.1%<br>Leu28 0.2%<br>Arg29 0.3%<br>Asn30 0.2%<br>Leu31 0.1%<br>Val32 0.2%<br>Arg34 0.4%	Lys12 0.1%<br>Ile13 0.5%<br>Gly14 0.1%<br>Lys15 0.2%<br>Glu16 0.6%<br>Phe17 0.5%<br>Arg19 0.2%<br>Ile20 0.2%<br>Gln22 0.5%<br>Arg23 0.2%<br>Ile24 0.1%<br>Asp26 1%<br>Phe27 1.3%<br>Leu28 0.8%<br>Arg29 0.4%<br>Asn30 0.7%<br>Leu31 0.3%<br>Val32 0.4%<br>Pro33 0.2%<br>Arg34 0.5%	Ile13 0.2%<br>Lys15 0.4%<br>Lys18 0.8%<br>Arg19 1.6%<br>Ile20 0.1%<br>Ile24 0.1%<br>Lys15 1.2%<br>Val21 0.1%<br>Gln22 0.2%<br>Arg23 2.3%<br>Phe27 0.2%<br>Leu28 0.1%<br>Arg29 0.7%<br>Asn30 0.7%<br>Leu31 0.3%<br>Val32 0.4%<br>Pro33 0.3%<br>Arg34 1%
N-methylnicotinamide + water	Lys10 0.3%<br>Arg19 0.1%<br>Gln22 0.1%<br>Lys25 0.3%<br>Phe27 0.1%<br>Asn30 0.1%	Leu1 0.1%<br>Leu2 0.3%<br>Gly3 0.1%<br>Phe6 0.1%<br>Ser9 0.1%<br>Lys10 0.7%<br>Glu16 0.2%<br>Arg19 1.3%<br>Arg23 0.2%<br>Lys25 0.1%<br>Asp26 0.1%<br>Phe27 0.3%<br>Leu28 0.6%<br>Arg29 0.1%<br>Asn30 0.6%<br>Pro33 0.1%<br>Arg34 0.3%	Arg7 0.5%<br>Lys10 0.1%<br>Gly14 0.1%<br>Lys15 0.1%<br>Lys18 0.5%<br>Arg19 0.2%<br>Arg23 0.2%<br>Ile24 0.2%<br>Arg29 0.1%<br>Leu31 0.1%<br>Arg34 0.1%
Isonicotinamide + water	Leu1 0.1%<br>Leu2 0.1%<br>Gly3 0.1%<br>Ser9 0.2%<br>Lys10 0.1%<br>Glu11 0.1%<br>Lys15 0.1%<br>Phe17 0.1%<br>Arg19 0.1%<br>Gln22 0.2%<br>Lys25 0.4%<br>Leu28 0.1%<br>Asn30 0.3%<br>Arg23 0.3%	Leu1 0.2%<br>Leu2 0.2%<br>Asp4 0.3%<br>Phe5 0.3%<br>Phe6 0.7%<br>Arg7 0.1%<br>Lys8 0.1%<br>Ser9 0.2%<br>Lys10 0.2%<br>Glu11 0.2%<br>Ile13 0.2%<br>Glu16 0.3%<br>Phe17 0.1%<br>Lys18 0.2%	Leu1 0.2%<br>Gly3 0.2%<br>Phe5 0.1%<br>Phe6 0.1%<br>Arg7 0.8%<br>Lys8 0.2%<br>Ser9 0.5%<br>Lys10 0.3%<br>Glu11 0.2%<br>Lys12 0.3%<br>Ile13 0.2%<br>Gly14 0.1%<br>Lys15 0.3%<br>Lys18 0.3%

	Arg34 0.4%	Ile20 0.1%<br>Gln22 0.1%<br>Arg23 0.4%<br>Ile24 0.1%<br>Lys25 0.1%<br>Asp26 0.1%<br>Phe27 0.8%<br>Leu28 0.1%<br>Asn30 0.7%<br>Leu31 0.3%<br>Val32 0.4%<br>Arg34 0.1%	Arg19 0.8%<br>Ile20 0.1%<br>Val21 0.3%<br>Gln22 0.5%<br>Arg23 1.3%<br>Lys25 0.5%<br>Leu28 0.1%<br>Arg29 0.8%<br>Asn30 0.4%<br>Val32 0.1%<br>Arg34 0.6%
Niacinamide + methanol	Leu1 0.1%<br>Leu2 0.1%<br>Gly3 0.1%<br>Phe5 0.2%<br>Arg7 0.3%<br>Ser9 0.6%<br>Lys10 0.3%<br>Lys12 0.5%<br>Lys15 0.5%<br>Lys18 0.4%<br>Arg19 0.4%<br>Gln22 0.3%<br>Arg23 0.5%<br>Lys25 0.3%<br>Arg29 0.1%<br>Asn30 0.1%<br>Arg34 0.2%	Leu1 0.1%<br>Leu2 0.1%<br>Gly3 0.1%<br>Asp4 0.8%<br>Phe5 0.2%<br>Lys8 0.4%<br>Ser9 0.2%<br>Lys10 0.5%<br>Glu11 2.2%<br>Ile13 0.1%<br>Gly14 0.1%<br>Lys15 0.3%<br>Glu16 0.5%<br>Phe17 0.2%<br>Lys18 0.1%<br>Arg19 0.2%<br>Val21 0.1%<br>Gln 22 2.5%<br>Arg23 0.4%<br>Asp26 1.3%<br>Phe27 0.9%<br>Leu28 0.1%<br>Arg29 0.4%<br>Asn30 1.1%<br>Leu31 0.1%<br>Val32 0.5%<br>Pro33 1%<br>Arg34 1.2%	Leu1 4.3%<br>Leu2 0.9%<br>Gly3 0.9%<br>Asp4 0.2%<br>Phe5 0.1%<br>Arg7 4.1%<br>Lys8 2.8%<br>Ser9 0.6%<br>Lys10 3.7%<br>Glu11 0.2%<br>Lys12 3.3%<br>Ile13 0.1%<br>Lys15 4.4%<br>Lys18 2.4%<br>Arg19 4.7%<br>Gln22 1.2%<br>Arg23 4.7%<br>Ile24 0.1%<br>Lys25 2.7%<br>Phe27 0.1%<br>Arg29 2.9%<br>Asn30 1.3%<br>Val32 0.1%<br>Arg34 4.5%
N-methylnicotinamide + methanol	Leu1 0.4%<br>Leu2 0.1%<br>Arg7 0.5%<br>Lys8 0.2%<br>Ser9 0.3%<br>Lys10 0.4%<br>Lys12 0.3%<br>Lys15 0.2%<br>Phe17 0.1%<br>Lys18 0.3%<br>Arg19 0.4%<br>Gln22 0.4%	Asp4 0.3%<br>Glu16 0.2%<br>Gln22 0.3%<br>Lys25 0.1%<br>Asp26 1.8%<br>Phe27 0.4%<br>Arg29 0.2%<br>Asn30 0.6%<br>Leu31 0.3%<br>Val32 0.1%<br>Arg34 0.8%	Leu1 1.1%<br>Leu2 0.3%<br>Gly3 0.2%<br>Phe6 0.2%<br>Arg7 2%<br>Lys8 1.1%<br>Ser9 0.1%<br>Lys10 0.9%<br>Lys12 1.7%<br>Lys15 1.2%<br>Lys18 0.8%<br>Arg19 2%

	Arg23 0.7%<br>Lys25 0.1%<br>Arg29 0.3%<br>Asn30 0.5%<br>Arg34 0.9%		Gln22 0.8%<br>Arg23 2.8%<br>Lys25 0.8%<br>Arg29 1.4%<br>Asn30 0.5%<br>Pro33 0.1%<br>Arg34 1.9%
Isonicotinamide + methanol	Gly3 0.1%<br>Phe5 0.1%<br>Arg7 0.1%<br>Lys8 0.1%<br>Ser9 0.2%<br>Lys10 0.3%<br>Lys15 0.2%<br>Lys18 0.1%<br>Gln22 0.3%<br>Arg23 0.2%<br>Lys25 0.2%<br>Arg29 0.3%<br>Asn30 0.3%<br>Arg34 0.4%	Leu1 0.2%<br>Leu2 0.1%<br>Gly3 0.2%<br>Asp4 0.6%<br>Arg7 0.1%<br>Ser9 0.4%<br>Lys10 0.4%<br>Glu11 0.8%<br>Lys12 0.1%<br>Gly14 0.1%<br>Lys15 0.2%<br>Glu16 0.7%<br>Phe17 0.1%<br>Gln22 0.2%<br>Arg23 0.1%<br>Lys25 0.2%<br>Asp26 0.9%<br>Phe27 0.2%<br>Leu28 0.1%<br>Arg29 0.5%<br>Asn30 0.3%<br>Leu31 0.7%<br>Val32 0.5%<br>Pro33 0.3%<br>Arg34 0.9%	Leu1 1.4%<br>Leu2 0.5%<br>Gly3 0.4%<br>Phe5 0.5%<br>Arg7 1.4%<br>Lys8 1.6%<br>Ser9 0.3%<br>Lys10 3.1%<br>Lys12 0.7%<br>Lys15 0.8%<br>Lys18 1.5%<br>Arg19 2.1%<br>Gln22 0.4%<br>Arg23 3.1%<br>Lys25 1.1%<br>Phe27 0.2%<br>Leu28 0.1%<br>Arg29 1.4%<br>Asn30 0.7%<br>Pro33 0.1%<br>Arg34 1.8%
Niacinamide + octanol	Leu1 0.2%<br>Leu2 0.4%<br>Phe5 0.1%<br>Arg7 1.3%<br>Lys8 9.3%<br>Ser9 6.3%<br>Lys10 1%<br>Lys12 8.6%<br>Ile13 0.1%<br>Lys15 31.3%<br>Phe17 0.1%<br>Lys18 53.6%<br>Arg19 2.8%<br>Ile20 0.6%<br>Val21 0.6%<br>Gln22 21.7%<br>Arg23 0.9%<br>Ile24 0.4%<br>Lys25 2.1%	Leu1 1.6%<br>Asp4 5.5%<br>Phe5 1.1%<br>Lys8 3.4%<br>Ser9 0.5%<br>Lys10 0.1%<br>Glu11 16.8%<br>Lys12 0.2%<br>Ile13 0.2%<br>Lys15 0.1%<br>Glu16 12.7%<br>Phe17 0.7%<br>Arg19 2.1%<br>Ile20 0.4%<br>Val21 0.1%<br>Gln22 49%<br>Ile24 0.2%<br>Lys25 0.5%<br>Asp26 5.1%	Leu1 22.9%<br>Leu2 9.6%<br>Gly3 3.6%<br>Asp4 1%<br>Phe5 0.2%<br>Phe6 0.4%<br>Arg7 25.6%<br>Lys8 41.5%<br>Ser9 4.8%<br>Lys10 41%<br>Lys12 87.8%<br>Ile13 0.2%<br>Lys15 51.9%<br>Glu16 0.2%<br>Lys18 53.1%<br>Arg19 74.6%<br>Ile20 0.7%<br>Val21 0.4%<br>Gln22 16%

	Arg29 1.9%<br>Asn30 0.7%<br>Leu31 0.1%<br>Val32 0.7%<br>Arg34 0.3%	Phe27 1.7%<br>Leu28 0.6%<br>Arg29 10.1%<br>Asn30 13.1%<br>Leu31 0.1%<br>Val32 33.3%<br>Pro33 2.5%<br>Arg34 7.4%	Arg23 47.1%<br>Lys25 54.1%<br>Phe27 1%<br>Leu28 0.3%<br>Arg29 31.6%<br>Asn30 13.3%<br>Leu31 0.6%<br>Val32 1.70%<br>Arg34 10.4%
N-methylnicotinamide + octanol	Leu1 4.9%<br>Leu2 2.3%<br>Gly3 3.1%<br>Asp4 0.1%<br>Phe5 0.2%<br>Phe6 0.1%<br>Arg7 11.9%<br>Lys8 2.2%<br>Ser9 22.5%<br>Lys10 1.6%<br>Lys12 3.9%<br>Lys15 11.50%<br>Lys18 2.2%<br>Arg19 1.2%<br>Gln22 6.2%<br>Arg23 1.3%<br>Lys25 2.7%<br>Asn30 0.4%<br>Leu31 0.3%<br>Val32 1.7%<br>Arg34 0.4%	Gln22 3%<br>Asp26 0.1%<br>Leu28 2.5%<br>Arg29 1.9%<br>Asn30 0.7%<br>Leu31 8.8%<br>Val32 0.4%<br>Pro33 0.1%<br>Arg34 3.2%	Leu1 15.8%<br>Leu2 9.9%<br>Gly3 12.8%<br>Phe5 0.1%<br>Phe6 0.3%<br>Arg7 19.1%<br>Lys8 71.3%<br>Ser9 16.6%<br>Lys10 11%<br>Lys12 97.1%<br>Ile13 0.1%<br>Lys15 18.4%<br>Lys18 15.5%<br>Arg19 25.9%<br>Val21 0.1%<br>Gln22 4.5%<br>Arg23 4.7%<br>Lys25 30.8%<br>Phe27 0.1%<br>Leu28 0.2%<br>Asn30 2%<br>Leu31 0.3%<br>Val32 2.8%<br>Pro33 0.3%<br>Arg34 9.1%
Isonicotinamide + octanol	Leu1 0.2%<br>Phe5 0.1%<br>Phe6 0.2%<br>Arg7 1.2%<br>Ser9 1.1%<br>Lys10 0.7%<br>Lys12 0.4%<br>Lys18 0.4%<br>Arg19 0.5%<br>Gln22 4.5%<br>Arg23 0.4%<br>Lys25 1.2%<br>Arg29 0.1%<br>Asn30 1.3%<br>Pro33 0.1%<br>Arg34 0.5%	Leu1 0.6%<br>Asp4 1.6%<br>Phe5 0.1%<br>Phe6 0.2%<br>Ser9 1%<br>Glu11 0.8%<br>Glu16 1%<br>Arg19 0.3%<br>Ile20 0.2%<br>Val21 0.2%<br>Gln22 1.7%<br>Arg23 0.2%<br>Lys25 0.2%<br>Asp26 2.1%<br>Phe27 0.1%<br>Arg29 3.1%	Leu1: 40%<br>Leu2: 2.8%<br>Gly3: 17.2%<br>Asp4: 0.5%<br>Phe5: 0.1%<br>Arg7: 11%<br>Lys8: 11%<br>Ser9: 4.3%<br>Lys10:34.7%<br>Lys12: 12.3%<br>Lys15:13.9%<br>Glu16:0.1%<br>Phe17:0.1%<br>Lys18:21.1%<br>Arg19:30.4%<br>Val21:0.1%

		Asn30 5.2%<br>Leu31 0.4%<br>Val32 2.1%<br>Pro33 4.6%<br>Arg34 25.7%	Gln22:23.6%<br>Arg23:6.3%<br>Lys25:14.9%<br>Asp26:0.3%<br>Phe27:0.1%<br>Arg29:12%<br>Asn30:4.9%<br>Arg34:15.4%
