# Supplementary material for: Small molecules enhance the potency of natural antimicrobial peptides
Source: Biophys J. 2021 Dec 24;121(3):491–501. doi: 10.1016/j.bpj.2021.12.029 (PMC8822605; doi:10.1016/j.bpj.2021.12.029)
Supplement: Document S2. Article plus supporting material [file mmc2.pdf]

Biophysical Journal
Article

Small molecules enhance the potency of natural antimicrobial peptides

Valeria Losasso,[1] Khushbu Agarwal,[2] Morris Waskar,[2] Amitabha Majumdar,[2] Jason Crain,[3,5] Martyn Winn,[1] and Michael Hoptroff[4,*]
[1]Science and Technology Facilities Council, Daresbury Laboratory, Sci-Tech Daresbury, Daresbury, UK; [2]Unilever Research and Development, Bangalore, India; [3]IBM Research Europe, Hartree Centre, Daresbury, UK; [4]Unilever Research and Development, Port Sunlight, UK; and [5]Department of Biochemistry, University of Oxford, Oxford, UK

ABSTRACT The skin-associated microbiome plays an important role in general well-being and in a variety of treatable skin conditions. In this regard, endogenous antimicrobial peptides have both a direct and indirect role in determining the composition of the microbiota. We demonstrate here that certain small molecular species can amplify the antimicrobial potency of naturally occurring antimicrobial peptides. In this study, we have used niacinamide, a form of vitamin B3 naturally found in foods and widely used in cosmetic skincare products, and two of its structural analogs, to investigate their cooperativity with the human antimicrobial peptide LL37 on the bacterium *Staphylococcus aureus*. We observed a clear synergistic effect of niacinamide and, to some extent, N-methylnicotinamide, whereas isonicotinamide showed no significant cooperativity with LL37. Adaptively biased molecular dynamics simulations using simplified model membrane substrates and single peptides revealed that these molecules partition into the headgroup region of an anionic bilayer used to mimic the bacterial membrane. The simulated effects on the physical properties of the simulated model membrane are well correlated with experimental activity observed in real biological assays despite the simplicity of the model. In contrast, these molecules have little effect on zwitterionic bilayers that mimic a mammalian membrane. We conclude that niacinamide and N-methylnicotinamide can therefore potentiate the activity of host peptides by modulating the physical properties of the bacterial membrane, and to a lesser extent through direct interactions with the peptide. The level of cooperativity is strongly dependent on the detailed chemistry of the additive, suggesting an opportunity to fine-tune the behavior of host peptides.

SIGNIFICANCE Antimicrobial peptides are widely found in nature and form a key component of the human innate immune system, deterring colonization by microbial pathogens through membrane disruption.
We show that small molecule additives can enhance the potency of these naturally occurring defense peptides, and we explore the molecular mechanisms responsible for this amplification using simplified model systems. Results obtained from a combination of experiment and computer simulation are presented for niacinamide, a well-known cosmetic ingredient widely used as an emollient, and its structural analogs.
This article provides novel insights indicating that, in addition to known effects on gene expression, niacinamide and related compounds cooperatively enhance the action of antimicrobial peptides through direct interactions with the lipid membrane and the peptide itself.

INTRODUCTION

It is increasingly recognized that consumer well-being issues such as body malodor (1), dandruff (2), and conditions such as atopic dermatitis (3) are commonly associated with imbalances in the skin microbiome. There is an emerging consensus that the Staphylococcal population of the skin microbiome, reflected in the ratios of *S. hominis* to *S. epidermidis* and *S. capitis* to *S. epidermidis* are implicated in axillary malodor and in scalp health, respectively. There is also increasing recognition of the link between high *Staphylococcus aureus* populations and human skin conditions such as atopic dermatitis (3,4).

S. aureus colonization of the human population is widespread, with approximately 30% of all humans carrying the organisms as a benign microbial inhabitant of the internal nasal cavity (5,6). However, colonization by *S. aureus* of the exposed skin of the face or body is commonly associated

Submitted July 22, 2021, and accepted for publication December 20, 2021.
*Correspondence: michael.hoptroff@unilever.com
Editor: Roland Winter.
https://doi.org/10.1016/j.bpj.2021.12.029
© 2021 Biophysical Society.
This is an open access article under the CC BY license (http://creativecommons.org/licenses/by/4.0/).

with negative pathologies including atopic dermatitis where the mean relative abundance of *S. aureus* has been reported as increasing to 65% during an atopic flare event compared to 1.1% in healthy controls (3).

The human body has evolved defenses that modulate the human-associated microbiome through mechanisms including the production of antimicrobial peptides (AMPs)—a family of small, endogenously produced compounds released through sweat and sebaceous secretions (7). They are a primitive form of defense mechanism and part of the innate immune system. AMPs bind to acidic phospholipids, which confer a net negative charge to bacterial membranes, leading to AMP aggregation and integration, leading to local membrane thinning (8). AMPs can target other structures or microbial processes, such as internal organelles, or they may inhibit enzyme activity or macromolecule synthesis (9). Keratinocytes, forming a large proportion of normal, healthy epidermal skin cells, produce AMPs, such as human cathelicidin LL-37, beta-defensins 2 and 3, and dermcidin, which contribute to the skin's ability to deter the overgrowth of undesirable micro-organisms (10). AMPs are expressed by keratinocytes either constitutively or are upregulated in response to microbial stimuli. Commensal and pathogenic staphylococci have been shown to activate different pathways in human keratinocytes, and commensals are able to amplify the innate immune response of keratinocytes to pathogens (11).

In the skin, the two best characterized families of AMPs are the defensins and cathelicidins. Defensins are packed in lamellar bodies within keratinocytes and released to the cell surface (12). Human beta defensin-2 is active against gram-negative bacteria, whereas beta defensin-3 kills both gram-negative and gram-positive bacteria such as *S. aureus* (13). Within the cathelicidin group, LL-37 has a broad activity spectrum and is reported to be effective against gram-positive and gram-negative bacteria and viruses (12). Other AMPs that also play a role in skin defense against pathogens include Psoriasin, which shows activity against *E. coli,* and RNase7, which has activity against *S. aureus* as well as gram-negative bacteria (12). In addition to their antimicrobial role, AMPs also function as immuno-modulators that could help supplement skin defenses.

Discovering how AMPs exert their antimicrobial effect and translating this insight into consumer products that work in partnership with natural defense peptides is important when identifying innovative and sustainable technologies for consumers. The observed target specificity suggests that AMPs are sensitive to the composition of the lipid membranes. This raises the possibility that AMP activity may be modulated by influencing lipid composition of the microbial target, rather than the more usual route of re-designing the AMP.

In principle, there may be several mechanisms by which small molecules can potentiate the activity of endogenous AMPs such as LL-37. Such potentiators could display a biological mechanism, for example, by increasing the biological expression of functional AMPs or by increasing their conversion from less antimicrobial pro-forms. Alternatively, they may participate directly in the antimicrobial activity, for example, by interacting with the AMP at a structural level or by an indirect physical mechanism such as destabilization of the bacterial membrane and enhancing the action of the AMP. It has been reported previously that small molecules, like niacinamide, that are not well-known as antimicrobials nonetheless give hygiene benefits by enhancing the expression level of AMPs in human tissue (1). However, no work to our knowledge has investigated the direct potentiation of the antimicrobial activity of AMPs through small molecules such as niacinamide.

This paper therefore seeks to explore the hypothesis that small molecules may amplify the potency of naturally occurring AMPs through mechanisms of physical interaction, in addition to the biological mechanisms previously reported (14). To precisely investigate such physical mechanisms, this work focused on in vitro and in silico models of AMP and potentiator interaction with bacterial membrane and has excluded the investigation with viable human cells.

Niacinamide is naturally found in foods, and it is widely used as a cosmetic skin care ingredient that has been also shown to mitigate against infection in mice (15) and to enhance AMPs in gut epithelial cells, in neutrophils (16), and in skin cells (14).

Here, we use computer simulations to investigate how single AMPs and candidate potentiator molecules interact with bacterial membranes at the molecular scale. This strategy seeks to demonstrate the rudimentary biophysical basis for potency amplification in model systems as a first step toward developing a fuller model of these processes that would include more realistic membrane compositions and the influence of peptide cooperativity.

Furthermore, we seek to explore correlations between the simulated model systems and microbial growth in a well-controlled single species in vitro model of microbial inactivation by LL37. To demonstrate the specificity of the interaction to bacterial membranes, similar in silico work was completed on model nonbacterial membranes.

Mammalian plasma membranes are asymmetric structures, with sphingomyelins and phosphatidylcholines primarily residing in their outer leaflet and aminophospholipids, phosphatidylserines, and phosphatidylethanolamines (PE) in their inner leaflet. The main components of bacterial membranes are, on the other hand, PE, phosphatidylglycerol, and cardiolipin (CL) (17). Notably, all bacterial membranes have anionic lipids in their composition such as PG and/or CL. The presence of these anionic lipids explains the selective toxicity of AMPs against bacteria but not against mammalian cells (18).

Simple phosphatidylcholine (POPC) and phosphatidylglycerol (POPG) models representing neutral mammalian membranes and negatively charged bacterial membranes

are routinely used in studies to easily represent differences between the two systems (see, for example, Zhang et al., Shahane et al., and Oliva et al. (19–21) and our previous work on AMPs (22). Therefore, we chose to use POPC and POPG, consistent with a recent simulation study on LL-37 (23).

MATERIALS AND METHODS

We assessed the following series of additives for their ability to act as potentiators of AMPs: niacinamide, isonicotinamide, and N-methylnicotinamide (Fig. 1). These are all naturally occurring analogs of vitamin B3. As an exemplar AMP, we chose the human peptide LL-37, with sequence LLGDFFRKSK–EKIGKEFKRI–VQRIKDFLRN–LVPRTES.

Microdilution assay

An overnight tryptic soy agar (TSA) plate culture of *S. aureus* ATCC 6538 was scraped and resuspended in 10 mM sodium phosphate buffer (pH 7.0 $\pm$ 0.2) to obtain a bacterial inoculum with cell number of 1 x 10^8 to 5 x 10^8 colony forming units per ml (cfu/mL). The inoculum was diluted to 1–5 x 10^6 cfu/mL before the assay. The assays were carried out in 96-well microtitre plates with a final volume of 300 μL. The additives (niacinamide, isonicotinamide, and N-methylnicotinamide) and AMP (LL37) and sodium phosphate buffer (100 mM, pH 7.0 $\pm$ 0.2, final 10 mM) were added to the wells, and the total volume was made up to 165 μL with sterile MilliQ water. One hundred and thirty-five μl of diluted bacterial inoculum was added to the wells, mixed gently, and incubated at 37 $\pm$ 0.1°C for 4 h. After the incubation period, aliquots were taken from the reaction mixtures and added to Dey-Engley (D/E) neutralizer broth. The neutralized samples were further diluted, plated onto TSA, and incubated for a minimum 24 h at 37°C. The viable bacteria form colonies on TSA plates after the incubation period that were counted to calculate the recovery (24,25).

Molecular dynamics simulations

We ran two independent 1-microsecond-long molecular dynamics (MD) simulations for 22 different systems. Of these, 18 systems consisted of one of the three additives in either a membrane environment or in solution. For the membrane simulations, 20 additive molecules were initially placed on a grid across a 10 $\times$ 10 nm patch of POPC or POPG membrane, positioned 5 Å below the membrane surface, corresponding to the average z coordinates of the upper leaflet phosphorus atoms (Fig. S1 *a* and *b*). These lipid-small molecule systems were simulated both on their own, to assess the impact of the additives on the membrane, and in presence of one LL-37 peptide. The peptide was added after 500 ns equilibration and placed in a parallel orientation to the membrane, just above the membrane surface, similarly to the setup of a previous study on POPG/POPC-LL37 complexes (23) (Fig. S1 *c* and *d*). For the solution simulations, 20 additive molecules were placed in a solvent box of volume ~1.3 x 10^6 Å^3 containing water, methanol, or octanol, and with one LL-37 peptide placed in the center of the box. The concentration of additives is small enough to prevent their self-aggregation—other than transient, mostly pairwise contacts (Fig. S2). The remaining four simulations were controls consisting of POPC or POPG membranes on their own or containing one LL-37 peptide, but in the absence of additives. Table 1 summarizes all the simulations performed and lists also the six systems used for free energy calculations (see next paragraph).

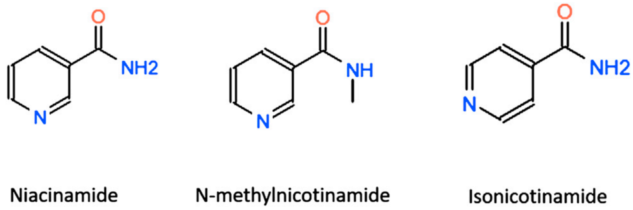

FIGURE 1 Additive molecules considered.

For simulations in solutions, we used the TIP3P model (26) for water and the CGenFF force field (27) for methanol and octanol. POPC and POPG membranes, composed of 294 and 324 lipids respectively, were described using the CHARMM36 force field for lipids (28). The structure of LL37 was taken from Protein Data Bank (ID 2K6O) and parameterized with CHARMM27 (29). Small molecules were generated using the ACEDRG program within the CCP4 suite (30) and then parameterized with GAFF force field (31) and AM1-BCC charges (32) through the Antechamber module in AMBER. Membranes were solvated with a 20-Å water layer on each side. Sodium counter-ions were used for charge neutralization in water and membrane simulations. Systems in membrane were equilibrated using the following protocol: 1) 5000 minimization steps, 2) 10 ns with harmonic constraints (1 kcal/mol/A^2) on protein and lipid heads, 3) 10 ns with harmonic constraints (1 kcal/mol/A^2) on protein only, and 4) 10 ns without constraints. Systems in solutions were equilibrated by following steps 1) and 3). Two independent replicas per system were simulated in production runs for 1 microsecond with constant temperature and pressure. All the simulations were carried out with the NAMD 2.9 software (33).

For the in-membrane systems, we analyzed the following properties: 1) instantaneous membrane thickness measured between the average phosphorus atom *z* coordinate for the upper and lower leaflets, 2) instantaneous area per lipid, 3) deuterium order parameter S_{CD}, a parameter typically derived in NMR experiments that reflects the orientational mobility of

TABLE 1 Summary of the systems studied

Simulation Type/time	Membrane/ solvent	Protein	Additive
2 x 1 μs unbiased	POPC	–	–
2 x 1 μs unbiased	POPC	LL37	–
2 x 1 μs unbiased	POPG	–	–
2 x 1 μs unbiased	POPG	LL37	–
2 x 1 μs unbiased	POPC	–	niacinamide
13 × 40 ns PMF	POPC	–	niacinamide
2 x 1 μs unbiased	POPC	–	N-methylnicotinamide
13 × 40 ns PMF	POPC	–	N-methylnicotinamide
2 x 1 μs unbiased	POPC	–	isonicotinamide
13 × 40 ns PMF	POPC	–	isonicotinamide
2 x 1 μs unbiased	POPG	–	niacinamide
13 × 40 ns PMF	POPG	–	niacinamide
2 x 1u μs unbiased	POPG	LL37	niacinamide
2 x 1 μs unbiased	POPG	–	N-methylnicotinamide
13 × 40 ns PMF	POPG	–	N-methylnicotinamide
2 x 1 μs unbiased	POPG	LL37	N-methylnicotinamide
2 x 1 μs unbiased	POPG	–	isonicotinamide
13 × 40 ns PMF	POPG	–	isonicotinamide
2 x 1 μs unbiased	POPG	LL37	isonicotinamide
2 x 1 μs unbiased	water	LL37	niacinamide
2 x 1 μs unbiased	methanol	LL37	niacinamide
2 x 1 μs unbiased	octanol	LL37	niacinamide
2 x 1 μs unbiased	water	LL37	N-methylnicotinamide
2 x 1 μs unbiased	methanol	LL37	N-methylnicotinamide
2 x 1 μs unbiased	octanol	LL37	N-methylnicotinamide
2 x 1 μs unbiased	water	LL37	isonicotinamide
2 x 1 μs unbiased	methanol	LL37	isonicotinamide
2 x 1 μs unbiased	octanol	LL37	isonicotinamide

each C-H bond along the aliphatic lipid tails and thus membrane fluidity, 4) the average tilt angle of all lipids with respect to the membrane normal, and 5) the time average of the mean-square displacement (MSD) of lipid molecules, a measure of lipid lateral mobility (34). All these properties were computed with the MEMBPLUGIN tool (35). For each system, data from the two replica simulations were combined, and the plots show a combined distribution or an appropriate average.

For all simulations including LL37 (in membrane or in solution), we analyzed the hydrogen bonds between small molecules and the protein, using as cutoffs 3.5 Å for donor-acceptor distance and 30° for donor-hydrogen-acceptor angle, and their transient unspecific contacts, defined with a maximum distance of 3 Å between any atom of the potentiator and any atom of the protein.

Free energy estimation

Potential of mean force (PMF) calculations were used to estimate the free energy barrier for each small molecule to penetrate the membrane, using the adaptive biasing force method as implemented in NAMD (36). The reaction coordinate was chosen as the distance between the center of mass of the potentiator and the surface of the membrane, defined as the instantaneous average of z coordinates of phosphorus atoms of the upper leaflet. The calculations start from the endpoint of the equilibration phase, and the reaction coordinate runs from the equilibrated position of the molecules to 10 Å below the membrane surface.

The reaction coordinate was decomposed into consecutive windows of size 1 Å, and each one of these was simulated for 40 ns with a force constant of 10 (kcal/mol)/Å^2 to confine the sampling within the window. This timescale was deemed sufficient for convergence based on a literature review of similar studies (22,37–39). As a further validation, we calculated the PMF every 10 ns for the example case of POPG + niacinamide, and found little difference between the curves after 30 and 40 ns (the convergence data is shown in Fig. S3).

RESULTS

Activity against *S. aureus* ($N = 2$)

The amplification of LL37 by each of the additives against a cell culture of *S. aureus* was evaluated using modified microdilution assays (24) as described in the Materials and Methods. The reactions were performed in a low-salt buffer as high concentrations of salts are known to be inhibitory to AMP activity. Fig. 2 shows the bacterial recovery rate averaged over four replicates taken from two independent experiments. Although niacinamide has no effect on the recovery rate by itself, it increases the measured activity of LL37 compared with LL37 alone. A similar but smaller effect is seen for N-methylnicotinamide. In the case of isonicotinamide, the effect is not significant at a 5% confidence threshold. Given the chemical similarity of the three additives, these differences are striking but reproducible (see Table S1 for individual results).

Interaction of additive molecules with model membranes

To explore the assay results at the molecular scale, we performed computer simulations of the relevant molecules in model membranes. We began by considering the interaction of niacinamide and its analogs on their own with a lipid bilayer to provide a reference point for subsequent simulations with AMP molecules included. We chose POPC and POPG as simple model membranes for human and bacterial cells, respectively, a model consistent with a recent simulation study on LL37 (23).

The association of molecules with lipid bilayers and subsequent insertion kinetics may encounter significant energy barriers that present challenges for conventional MD methods to sample correctly. Here we use adaptive-biasing-force simulations to overcome barriers in the free energy landscape. Integration of the average force along a chosen reaction coordinate gives a measure of the free energy of insertion for these molecules through the PMF; see Fig. 3. Insertion is generally unfavorable for niacinamide and its derivatives, except around the headgroup region, which is consistent with negative logP values determined experimentally (e.g., as obtained from PubChem entries). Notably, the niacinamide preferential localization is in contrast with the behavior reported in PMF studies for a similar molecule, thymol, which shows instead a clear propensity to insert into the hydrophobic interior of a dipalmitoylphosphatidylcholine (DPPC) model membrane (37). This can be explained by the higher polarity of niacinamide due to the presence of both the pyridine nitrogen and the amide group as substituents.

All molecules show a local minimum in the free energy between 2 and 5 Å below the membrane surface. For each

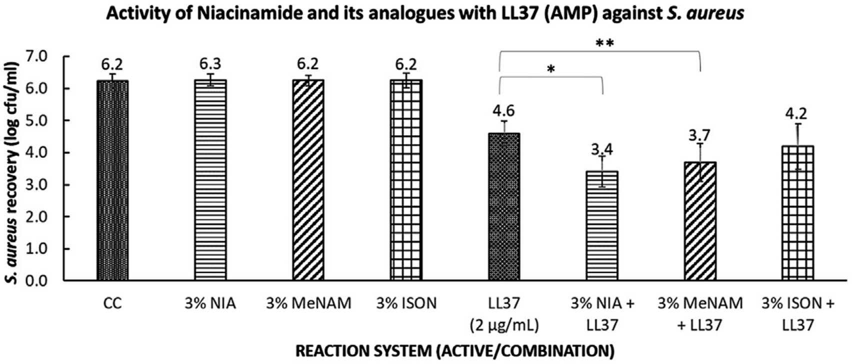

FIGURE 2 Microdilution Assay of niacinamide and its analogs against *S. aureus*. Niacinamide shows significant amplification of LL37 potency, and this synergy is present at reduced levels with N-methylnicotinamide, whereas no significant synergy was observed with isonicotinamide. Key: CC = culture control (no treatment), NIA = niacinamide, MeNAM = N-methylnicotinamide, ISON = isonicotinamide, LL37 = cathelicidin antimicrobial peptide (AMP), used at 2 microgram per ml concentration. Data is from two independent repeat experiments, each comprising two replicates. Error bars are SE of mean. The symbols * and ** correspond to $p < 0.05$ (one-tailed t-test).

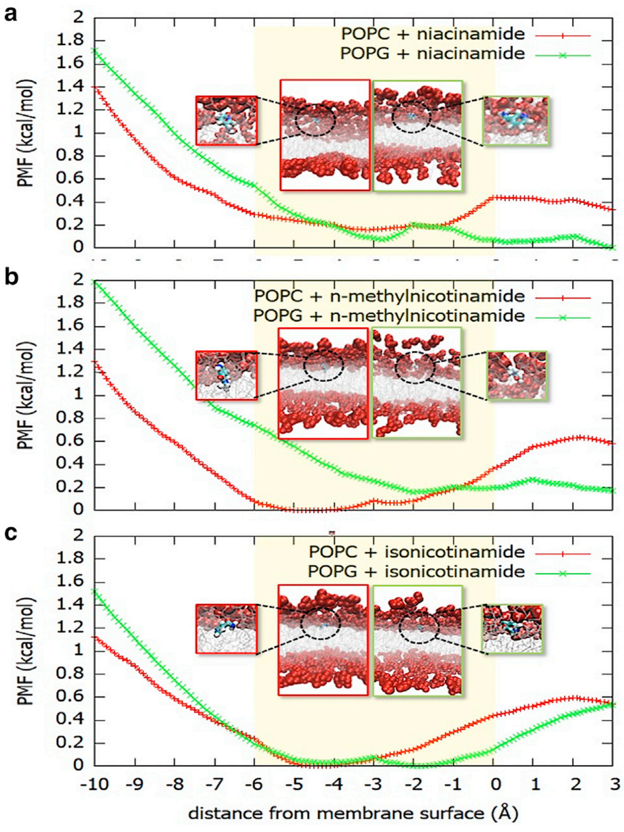

FIGURE 3 PMF showing the free energy for insertion of (*a*) niacinamide, (*b*) N-methylnicotinamide, and (*c*) isonicotinamide in POPC and POPG model membranes. The PMF is given as a function of the distance from the membrane surface, defined as the average *z* coordinate of the upper leaflet phosphorus atoms. The lipid headgroups lie roughly in the region –6 Å to 0 (yellow boxes), and the center of the bilayer is at −20 Å (not shown). The location of the molecules in the membrane at the free energy minima are shown in the boxes (red frame for POPC, green frame for POPG; headgroups are shown as red spheres, lipid tails as silver lines, additives as CPK-colored sticks). Unbiased simulations were used to confirm the PMF results and provide more detail on molecular interactions. Additive molecules were initialized at a depth of 5 Å in the membrane, in the region of the free energy minima shown by the PMF curves. We examined all three additives and give example results for the case of niacinamide.

molecule the process of reaching the surface is more favorable for negatively charged POPG than for POPC, as a result of the polarity of the substituent groups. Thus, the free energy surface for niacinamide and N-methylnicotinamide is relatively flat from 3 Å on either side of the POPG membrane surface.

An analysis of the membrane thickness and the area per lipid across the simulated patch shows that niacinamide induces thinning and stretching of the POPG membrane (Fig. S4 *a* and *b*); however, it has little effect on POPC membranes. The deuterium order parameter (S_{CD}) shows that it induces disorder along the lipid tails for a POPG membrane, whereas the effect is not observed for POPC (Fig. S4 *c*). The lipid tail disorder is also shown by an increase in lipid tilt angle (averaged over all lipid molecules, Fig. S4 *d*) with respect to the membrane normal when niacinamide is simulated in combination with POPG (Fig. S4 *e*). Finally, niacinamide increases POPG membrane fluidity, as indicated by the ensemble average of time-averaged MSDs (TAMSD) as a function of the measurement time (Fig. S4 *f*).

Focusing on the POPG membrane, a collective analysis of niacinamide together with its derivatives shows that they affect membrane properties in the order niacinamide > N-methylnicotinamide > isonicotinamide (Fig. 4). Following this order, all three additives decrease POPG thickness (Fig. 4 *a*) and increase area per lipid (Fig. 4 *b*), although niacinamide displays some variability between the two simulation replicas, resulting in a bimodal distribution. The three molecules all decrease the S_{CD} order parameter, with small differences among them (Fig. 4 *c*). However, the lipid tilt angle shows a significant increase in tail disorder in the presence of niacinamide compared with its derivatives and with pure POPG (Fig. 4 *d*).

Fig. 4 *e* shows the ensemble average (over all lipid molecules) of the time-averaged MSDs for POPG as a function of the measurement time. These values suggest that the lipid mobility is also higher in the presence of niacinamide. This quantity is indeed linked to lateral diffusion coefficients for lipids (34), and it shows the effect of the presence of the additives in the first few hundreds of nanoseconds of simulation; the long time behavior, with all systems close to a value of ~0.4 $Å^2$/ns, although not fully converged, is in agreement with experimental values reported for POPG lateral diffusion coefficients (38). These results are consistent with a mechanism where additives perturb the membrane on a short timescale (<1 μs), which is however sufficient for AMPs to exert their action, as demonstrated in Fig. 5 *a*.

In summary, niacinamide and its analogs partition into the headgroup region of POPC or POPG bilayers. All three additives have a clear effect on the physical properties of the POPG membrane, although the extent varies, and the effect on POPC membranes is less clear. These results suggest that a suitable concentration of additives could alter how anionic membranes interact with AMPs.

Stability and orientation of LL37 in model membranes

We next considered the interaction of a single AMP, as exemplified by LL-37, with the POPC and POPG model membranes in the absence of additive molecules. We analyzed the ability of the peptide to enter and deform the lipid bilayers, as well as the conformational changes of the peptide itself.

Starting from an initial position above the membrane surface, LL-37 locates in the headgroup region of POPG membranes, and it does not bind POPC membranes and remains in aqueous solution, as shown by the distribution of distances to the membrane surface (Fig. 5 *a*), and in agreement with the

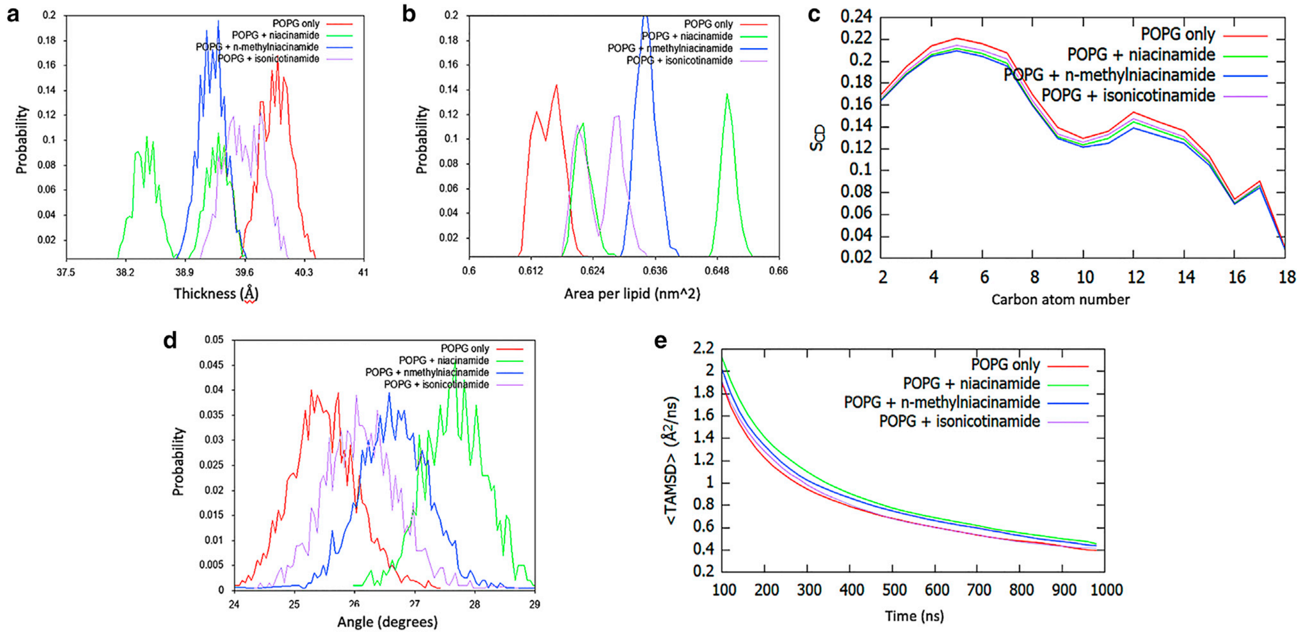

FIGURE 4 Analysis of unbiased simulations for niacinamide and its derivatives in complex with POPG membranes: (*a*) distribution of instantaneous membrane thickness values, (*b*) distribution of area-per-lipid values, (*c*) S_{CD} order parameter, (*d*) distribution of lipid tilt angles, and (*e*) POPG time-averaged MSD.

binding pattern described by Zhao et al. (23). Consistently with all helical AMPs (39), and with the previous study on LL37 (23), it preserves most of its secondary structure when bound to POPG (Fig. 5 *b*), and it shows partial disruption of its helical configuration in the presence of POPC (Fig. 5 *b*), where the water environment contributes to the unfolding of the region between residues 8 and 15 (Fig. 5 *c*).

In binding POPG, LL-37 also affects the physical properties of the membrane (Fig. S5). In particular, it reduces its average thickness (Fig. S5 *a*) and increases its disorder and fluidity (Fig. S5 *c–e*), although it does not significantly affect its area per lipid (Fig. S5 *b*). In contrast, there is no significant effect on POPC membranes. The decrease of POPG membrane thickness in the presence of LL-37 as opposed to POPC is in agreement with the previously published study of POPC/POPG-LL37 complexes (23), which used a similar system setup and simulation protocol but a different force field (GROMOS 53a6 as opposed to CHARMM36).

Interaction of additive molecules with LL37

We next considered direct interactions of additive molecules with LL-37. Given the failure of LL-37 to insert into the POPC membrane, we focus here on the observations for the POPG membrane system. Simulation of model POPG membranes with both additives and LL-37 included revealed transient contacts (Table S2; Fig. 6). However, we did not observe any stable complexes between the additive molecules and LL-37 in the membrane environment.

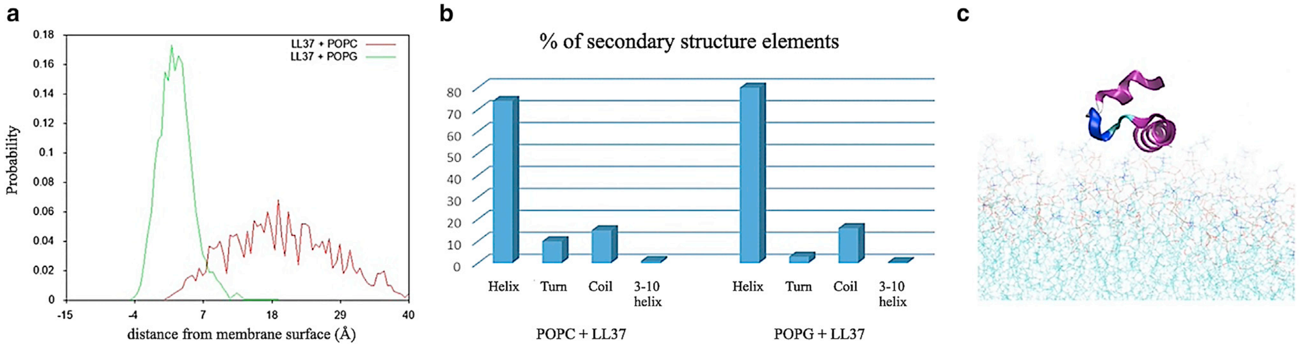

FIGURE 5 Simulations for LL37 in complex with POPG and POPC membranes: (*a*) Distribution of distances between LL-37 center of mass and POPG/POPC membrane surfaces. (*b*) Percentage of secondary structure elements in the LL-37 structure in complex with POPG or POPC. (*c*) Snapshot of LL-37 in complex with POPC (purple = alpha helix; blue = coil).

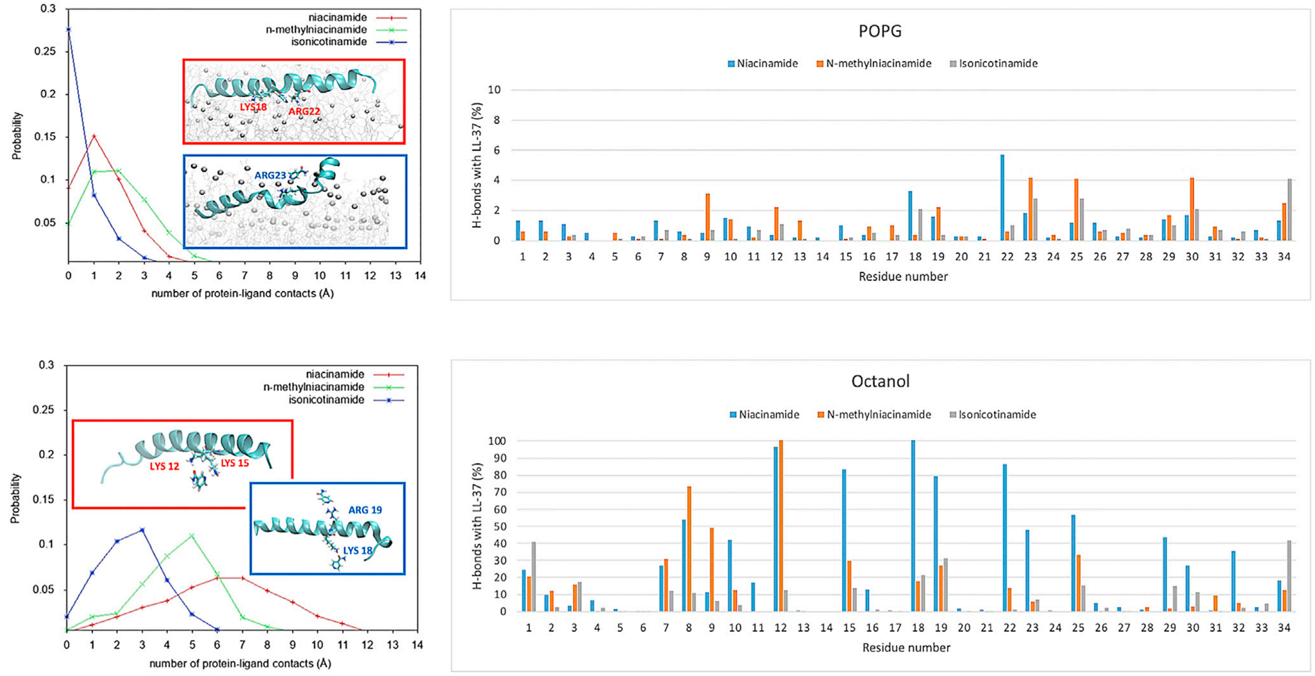

FIGURE 6 Distribution of contacts for niacinamide and its derivatives with LL-37 in membrane (top) and octanol (bottom). Representative snapshots for niacinamide and isonicotinamide binding are shown in red and blue boxes, respectively.

The lack of contacts may be due to poor sampling in the MD simulation, so we also considered additives and LL-37 in solution. To mimic different environments and their effect on electrostatic interactions, we considered three solvents: water, methanol, and octanol. Although these simulations do not represent the membrane environment, they allow us to collect better statistics on potential direct interactions.

In water, all niacinamide derivatives show a few transient contacts with LL-37 (Fig. S6). In contrast, in hydrophobic environments, we observe different effects. Whereas in the membrane the largest number of contacts were observed for N-methylnicotinamide (Fig. 6), niacinamide shows more contacts in hydrophobic solvents (methanol and octanol, Figs. S6 and 6). Interestingly, isonicotinamide showed few contacts in all solvents, despite being an isomer of niacinamide. Isomerization leads to a charge redistribution in the conjugated system (Fig. S7 *a*). As a control, RESP atomic charges (40) obtained by fitting with the electrostatic potential calculated at the B3LYP/HF-6-31G* level with Gaussian 16 (41) are reported in Fig. S7 *b* and show a similar redistribution upon isomerization. Based on the current observation, the position of the ring N in niacinamide permits a particular type of bonding that allows for more contacts with LL-37 to be formed, which is reflected in a different binding orientation to LL-37 (Fig. S7 *c*) and the possibility of forming simultaneous multiple interactions (Fig. S8).

We also analyzed the formation of hydrogen bonds between additives and LL-37 in the four different contexts, to investigate the chemical features underlying the differences between niacinamide and its analogs, in particular its isomer isonicotinamide. We did not observe stable hydrogen bonds in the membrane systems or in water (Table S2), so we used hydrophobic solvents to mimic the membrane environment while allowing for a faster diffusion.

In methanol, we start observing more persistent hydrogen bonds between the amide oxygen of niacinamide and positively charged residues of the central region of LL-37 (Lys12, Lys15, Lys18, Arg19, Arg23), which become less frequent in the case of N-methylnicotinamide and isonicotinamide. N-methylnicotinamide tends to bind more efficiently the region of the LL-37 residues 7–12 (Fig. 6). However, clearer differences among the three analogs are observed in octanol, where niacinamide shows more overall contacts with respect to its derivatives, especially isonicotinamide. Niacinamide forms stable and sustained contacts between its polar atoms and a small group of residues (Lys12, Lys15, Lys18, Arg19, Gln22, Arg23), which are also found at a lower extent in N-methylnicotinamide but at a much lower extent in isonicotinamide (Table S2, Fig. 6). In particular, niacinamide amide oxygen and ring nitrogen can form simultaneous interactions with Lys12 and Lys15 side chains as well as with Gln22 and Lys18 side chains, respectively (Fig. S8). Therefore, the nature and position of the substituents on the niacinamide ring play a crucial role in the preferential binding of this additive to LL-37 when compared with N-methylnicotinamide and isonicotinamide, by allowing niacinamide to form multiple bonds with the peptide. Moreover, consistently in both

POPG and octanol, niacinamide forms the highest number of contacts with Lys18 and Gln22 (Fig. 6). These two residues belong to the LL-37 active core (residues 17–29), which was shown to represent the key region for the biological functions of the peptide (42,43) and to be able to self-assemble into protein fibril of densely packed helices that can enhance antimicrobial activity (44). Therefore, niacinamide is potentially able to act on peptide regions crucial for its function.

Combined effect

Finally, we explored the effect on POPG membrane properties of the combined presence of additives and LL37 compared with a control membrane in complex with LL37 only. The results can also be compared with the membrane properties in the presence of the additives only, shown in Fig. 4 *a–d* and replotted in Fig. 7.

In general, for niacinamide + LL-37, we see that thickness decreases, area per lipid increases, and lipid order (measured by S_{CD} parameter and lipid tilt angle) decreases, compared with the pure membrane or either component individually. These effects can also be seen in the other additives to a lesser extent, and we can again place the analogs in the order niacinamide > N-methylnicotinamide > isonicotinamide (Fig. 7). In particular, only niacinamide shows a strongly cooperative effect with LL-37, wherein the small molecule and AMP in combination impact membrane properties to a greater extent than either one individually (Table S2).

DISCUSSION

Our experimental assays have revealed that some small molecule additives can potentiate the activity of the naturally occurring endogenous skin AMP LL-37 against *S. aureus*, an organism closely associated with atopic dermatitis (3,4,45,46). Common cosmetic ingredients like niacinamide could potentially work with the innate defenses of the skin and provide enhanced protection against pathogens such as *S. aureus*. This understanding may be expanded to other harmful strains such as MRSA that cause severe disease and need to be controlled. There are other benefits from niacinamide reported in the literature, including boosting of antimicrobial peptide expression (14), and the synergy mechanisms reported here could work along with them. Taken together, our data demonstrates how a molecule such as niacinamide, which is not inherently antimicrobial, may enhance hygiene benefits by potentiating the body's natural defenses by a two-pronged mechanism that includes increasing the number of AMPs (14) and, as reported here, by potentiating their activity against pathogens. Such multifunctional technologies offer benefits not just to the host, but also keep undesirable microbes in check.

Using molecular simulations of these molecules and a model membrane, we have shown that additives can alter lipid bilayer properties such as thickness, area per lipid, and acyl tail disorder, and thereby may render the membrane more susceptible to AMPs. These effects are much clearer for the anionic POPG bilayer, suggesting some specificity

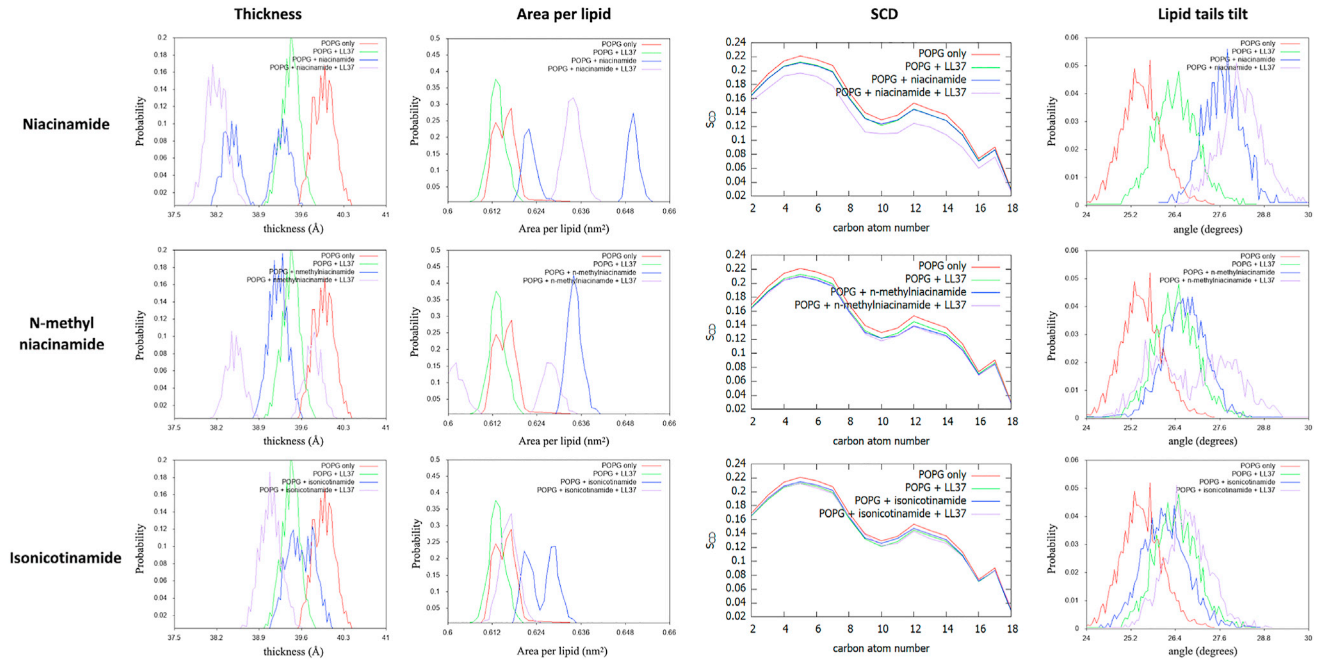

FIGURE 7 Effect on the membrane structure of the three additives in combination with LL37, as measured by membrane thickness, area per lipid, S_{CD} order parameter, and lipid tilt angle.

to the target membrane. Although POPG is often used to represent negatively charged membranes, either alone (23,47) or as part of a binary mixture (48), this is a simple model designed to highlight trends. Real bacterial membranes have a highly variable composition, depending on species and environmental conditions (49) and realistic modeling of a specific membrane is beyond the scope of the current study. Future work could consider other important classes of lipid, such as cardiolipins (17), which have been implicated in resistance to AMP's (50), or models of asymmetric membranes (51).

We have also shown that the additives can interact directly with AMP molecules. For simplicity, we considered a single LL-37 molecule, embedded in both membrane and solution environments, and observed both transient and stable contacts. The mode of action of LL-37 (and other AMPs) is usually assumed to proceed via pore formation, involving several peptides in a barrel or toroidal pore (52), although other mechanisms may also be involved. For example, based on several experimental probes applied to model membranes, Majewska et al. (53) argue for a "carpet" mechanism of LL-37. Our results suggest that small molecule additives may influence the mode of action of LL-37 through interactions with individual peptides, though the final effect on activity is likely to be dependent on the precise mechanism.

A significant outcome of our study is that there is a large variation between the three additives studied, even though they are all vitamin B3 structural analogs. Niacinamide partitions into the headgroup region of the bilayer. Although it does not fully permeate, it is still observed to have an effect on membrane physical properties. This is an example where traditional metrics, such as logP, can be misleading. It is also observed to make transient contacts with LL-37 both in solution and in the membrane, suggesting a direct synergistic effect. More stable interactions, including specific hydrogen bonding, are observed in hydrophobic solvents that are often used to represent the membrane interior. Although we do not observe niacinamide partitioning into the interior, this may be relevant in more complex membranes or upon AMP-led perturbation.

Isonicotinamide, although an isomer of niacinamide, is observed experimentally not to have a significant synergy with LL-37. Simulations support this result, in terms of its effect on the physical properties of model membranes and the lack of interaction with LL-37. Although some effects are seen in simulation, these are less clear-cut than for niacinamide and may not be significant physiologically. N-methylnicotinamide presents an interesting case. Although most metrics show an intermediate effect, it exhibits a higher number of contacts with LL-37 in the membrane (Fig. 6 *a*). This could imply a slightly different balance between the two proposed modes of action, i.e., destabilization of the membrane versus direct interaction with LL-37.

CONCLUSION

Our study has elucidated the mechanisms by which subtle changes in chemical structure influence observed cooperativity between certain small molecules and natural peptides leading to potency amplification. Further research would be valuable to determine how these insights apply to other small molecules and to optimize the potentiation of AMPs. Although the roles of peptide sequence and membrane composition are frequently considered in AMP research, the influence of small molecules present as metabolites or as external additives has not been considered before. This adds a further element to the subtle balance of factors involved in AMP action, some of which may complicate the interpretation of in vivo experiments, but which also presents opportunities for intervention.

Future studies may focus on screening for other potentiators, as an alternative strategy to screening for novel bioactives. In silico studies can help by screening for promising candidates, and the protocols used here may be useful. The effect of additives on the lipid bilayer requires an explicit membrane model, but changes in the physicochemical properties of the bilayer are readily apparent from unbiased simulations. Octanol is often used, both experimentally and computationally, as a simple mimetic of the bilayer interior, and here we have seen that it can reveal possible binding modes between additives and AMPs. Thus, a combination of simple computational techniques could reveal further potentiators. Of course, in the case of specific microbial targets, more sophisticated modeling is also possible.

Ultimately, this work enhances our understanding of how the activity of naturally occurring skin AMPs may be impacted by small molecules and thereby opens opportunities for applications related to their enhanced in situ efficacy and modulation by topical treatments.

SUPPORTING MATERIAL

Supporting Material can be found online at https://doi.org/10.1016/j.bpj.2021.12.029.

AUTHOR CONTRIBUTIONS

Designed research: J.C., M. Winn, A.M., M.H. Supervised research: J.C., M. Winn. Acquired funding: J.C., M. Winn. Managed project: M.H. Performed research: V.L., K.A., M. Waskar. Analyzed data: V.L., K.A., M. Waskar. Wrote original draft: V.L., M. Waskar, A.M., M.H. Reviewed/edited manuscript: J.C., M. Winn, M. Waskar, A.M., M.H.

ACKNOWLEDGMENTS

This work was supported by the Science and Technology Facilities Council and the Hartree National Centre for Digital Innovation program, funded by the Department for Business, Energy & Industrial Strategy.

REFERENCES

1. Rudden, M., R. Herman, …, G. H. Thomas. 2020. The molecular basis of thioalcohol production in human body odour. *Sci. Rep.* 10:12500. https://doi.org/10.1038/s41598-020-68860-z.
2. Grimshaw, S. G., A. M. Smith, …, B. Murphy. 2019. The diversity and abundance of fungi and bacteria on the healthy and dandruff affected human scalp. *PLoS One.* 14:e0225796. https://doi.org/10.1371/journal.pone.0225796.
3. Kong, H. H., J. Oh, …, J. A. Segre. 2012. Temporal shifts in the skin microbiome associated with disease flares and treatment in children with atopic dermatitis. *Genome Res.* 22:850–859. https://doi.org/10.1101/gr.131029.111. https://pubmed.ncbi.nlm.nih.gov/22310478.
4. Meylan, P., C. Lang, …, S. Christen-Zaech. 2017. Skin colonization by Staphylococcus aureus precedes the clinical diagnosis of atopic dermatitis in infancy. *J. Invest. Dermatol.* 137:2497–2504. https://doi.org/10.1016/j.jid.2017.07.834.
5. Sakr, A., F. Brégeon, …, O. Blin. 2018. Staphylococcus aureus nasal colonization: an update on mechanisms, epidemiology, risk factors, and subsequent infections. *Front. Microbiol.* 9:2419. https://doi.org/10.3389/fmicb.2018.02419.
6. Krismer, B., C. Weidenmaier, …, A. Peschel. 2017. The commensal lifestyle of Staphylococcus aureus and its interactions with the nasal microbiota. *Nat. Rev. Microbiol.* 15:675–687. https://doi.org/10.1038/nrmicro.2017.104.
7. Gläser, R., J. Harder, …, J. M. Schröder. 2005. Antimicrobial psoriasin (S100A7) protects human skin from Escherichia coli infection. *Nat. Immunol.* 6:57–64. https://doi.org/10.1038/ni1142.
8. Wiesner, J., and A. Vilcinskas. 2010. Antimicrobial peptides: the ancient arm of the human immune system. *Virulence.* 1:440–464. https://doi.org/10.4161/viru.1.5.12983.
9. Lei, J., L. Sun, …, Q. He. 2019. The antimicrobial peptides and their potential clinical applications. *Am. J. Transl. Res.* 11:3919–3931.
10. Rieg, S., S. Seeber, …, B. Schittek. 2006. Generation of multiple stable dermcidin-derived antimicrobial peptides in sweat of different body sites. *J. Invest. Dermatol.* 126:354–365. https://doi.org/10.1038/sj.jid.5700041.
11. Wanke, I., H. Steffen, …, B. Schittek. 2011. Skin commensals amplify the innate immune response to pathogens by activation of distinct signaling pathways. *J. Invest. Dermatol.* 131:382–390. https://doi.org/10.1038/jid.2010.328.
12. Clausen, M. L., and T. Agner. 2016. Antimicrobial peptides, infections and the skin barrier. *Curr. Probl. Dermatol.* 49:38–46. https://doi.org/10.1159/000441543.
13. Chen, X., F. Niyonsaba, …, H. Ogawa. 2005. Synergistic effect of antibacterial agents human beta-defensins, cathelicidin LL-37 and lysozyme against Staphylococcus aureus and Escherichia coli. *J. Dermatol. Sci.* 40:123–132. https://doi.org/10.1016/j.jdermsci.2005.03.014.
14. Mathapathi, M. S., P. Mallemalla, …, A. Majumdar. 2017. Niacinamide leave-on formulation provides long-lasting protection against bacteria in vivo. *Exp. Dermatol.* 26:827–829. https://doi.org/10.1111/exd.13285.
15. Kyme, P., N. H. Thoennissen, …, G. Y. Liu. 2012. C/EBPε mediates nicotinamide-enhanced clearance of Staphylococcus aureus in mice. *J. Clin. Invest.* 122:3316–3329. https://doi.org/10.1172/jci62070.
16. Hashimoto, T., T. Perlot, …, J. M. Penninger. 2012. ACE2 links amino acid malnutrition to microbial ecology and intestinal inflammation. *Nature.* 487:477–481. https://doi.org/10.1038/nature11228.
17. Lopes, S. C., C. S. Neves, …, P. Gameiro. 2012. Improved model systems for bacterial membranes from differing species: the importance of varying composition in PE/PG/cardiolipin ternary mixtures. *Mol. Membr. Biol.* 29:207–217. https://doi.org/10.3109/09687688.2012.700491.
18. Hale, J. D., and R. E. Hancock. 2007. Alternative mechanisms of action of cationic antimicrobial peptides on bacteria. *Expert Rev. Anti Infect. Ther.* 5:951–959. https://doi.org/10.1586/14787210.5.6.951.
19. Zhang, A. H., I. A. Edwards, …, M. Mobli. 2019. Elucidating the lipid binding properties of membrane-active peptides using cyclised nanodiscs. *Front. Chem.* 7:238. https://doi.org/10.3389/fchem.2019.00238.
20. Shahane, G., W. Ding, …, M. Orsi. 2019. Physical properties of model biological lipid bilayers: insights from all-atom molecular dynamics simulations. *J. Mol. Model.* 25:76. https://doi.org/10.1007/s00894-019-3964-0.
21. Oliva, R., P. Del Vecchio, …, L. Petraccone. 2019. Membrane disintegration by the antimicrobial peptide (P)GKY20: lipid segregation and domain formation. *Phys. Chem. Chem. Phys.* 21:3989–3998. https://doi.org/10.1039/c8cp06280c.
22. Losasso, V., Y. W. Hsiao, …, J. Crain. 2019. Modulation of antimicrobial peptide potency in stressed lipid bilayers. *Phys. Rev. Lett.* 122:208103. https://doi.org/10.1103/PhysRevLett.122.208103.
23. Zhao, L., Z. Cao, …, Y. Zhou. 2018. Molecular dynamics simulations of human antimicrobial peptide LL-37 in model POPC and POPG lipid bilayers. *Int. J. Mol. Sci.* 19. https://doi.org/10.3390/ijms19041186.
24. Murakami, M., T. Ohtake, …, R. L. Gallo. 2002. Cathelicidin anti-microbial peptide expression in sweat, an innate defense system for the skin. *J. Invest. Dermatol.* 119:1090–1095. https://doi.org/10.1046/j.1523-1747.2002.19507.x.
25. Sahly, H., S. Schubert, …, R. Podschun. 2003. Burkholderia is highly resistant to human Beta-defensin 3. *Antimicrob. Agents Chemother.* 47:1739–1741. https://doi.org/10.1128/aac.47.5.1739-1741.2003.
26. Jorgensen, W. L., J. Chandrasekhar, …, M. L. Klein. 1983. Comparison of simple potential functions for simulating liquid water. *J. Chem. Phys.* 79:926–935. https://doi.org/10.1063/1.445869.
27. Vanommeslaeghe, K., E. Hatcher, …, A. D. Mackerell, Jr. 2010. CHARMM general force field: a force field for drug-like molecules compatible with the CHARMM all-atom additive biological force fields. *J. Comput. Chem.* 31:671–690. https://doi.org/10.1002/jcc.21367.
28. Klauda, J. B., R. M. Venable, …, R. W. Pastor. 2010. Update of the CHARMM all-atom additive force field for lipids: validation on six lipid types. *J. Phys. Chem. B.* 114:7830–7843. https://doi.org/10.1021/jp101759q.
29. MacKerell, A. D., D. Bashford, …, M. Karplus. 1998. All-atom empirical potential for molecular modeling and dynamics studies of proteins. *J. Phys. Chem. B.* 102:3586–3616. https://doi.org/10.1021/jp973084f.
30. Long, F., R. A. Nicholls, …, G. N. Murshudov. 2017. AceDRG: a stereochemical description generator for ligands. *Acta Crystallogr. D Struct. Biol.* 73:112–122. https://doi.org/10.1107/s2059798317000067.
31. Wang, J., R. M. Wolf, …, D. A. Case. 2004. Development and testing of a general amber force field. *J. Comput. Chem.* 25:1157–1174. https://doi.org/10.1002/jcc.20035.
32. Jakalian, A., D. B. Jack, and C. I. Bayly. 2002. Fast, efficient generation of high-quality atomic charges. AM1-BCC model: II. Parameterization and validation. *J. Comput. Chem.* 23:1623–1641. https://doi.org/10.1002/jcc.10128.
33. Phillips, J. C., R. Braun, …, K. Schulten. 2005. Scalable molecular dynamics with NAMD. *J. Comput. Chem.* 26:1781–1802. https://doi.org/10.1002/jcc.20289.
34. Giorgino, T. 2019. Computing diffusion coefficients in macromolecular simulations: the diffusion coefficient tool for VMD. *J. Open Source Softw.* 4:1698. https://doi.org/10.21105/joss.01698.
35. Guixà-González, R., I. Rodriguez-Espigares, …, J. Selent. 2014. MEMBPLUGIN: studying membrane complexity in VMD. *Bioinformatics.* 30:1478–1480. https://doi.org/10.1093/bioinformatics/btu037.
36. Hénin, J., G. Fiorin, …, M. L. Klein. 2010. Exploring multidimensional free energy landscapes using time-dependent biases on collective variables. *J. Chem. Theor. Comput.* 6:35–47. https://doi.org/10.1021/ct9004432.
37. Ferreira, J. V., T. M. Capello, …, L. Caseli. 2016. Mechanism of action of thymol on cell membranes investigated through lipid Langmuir monolayers at the air-water interface and molecular simulation. *Langmuir.* 32:3234–3241. https://doi.org/10.1021/acs.langmuir.6b00600.

38. Saleem, Q., A. Lai, …, P. M. Macdonald. 2012. Lateral diffusion of bilayer lipids measured via (31)P CODEX NMR. *Chem. Phys. Lipids.* 165:721–730. https://doi.org/10.1016/j.chemphyslip.2012.08.001.

39. Dathe, M., and T. Wieprecht. 1999. Structural features of helical antimicrobial peptides: their potential to modulate activity on model membranes and biological cells. *Biochim. Biophys. Acta.* 1462:71–87. https://doi.org/10.1016/s0005-2736(99)00201-1.

40. Bayly, C. I., P. Cieplak, …, P. A. Kollman. 1993. A well-behaved electrostatic potential based method using charge restraints for deriving atomic charges: the RESP model. *J. Phys. Chem.* 97:10269–10280. https://doi.org/10.1021/j100142a004.

41. Frisch, M. J., G. W. Trucks, …, H. B. Schlegel. 2016. Gaussian 16, Revision C.01. Gaussian, Inc., Wallingford, CT.

42. Li, X., Y. Li, …, G. Wang. 2006. Solution structures of human LL-37 fragments and NMR-based identification of a minimal membrane-targeting antimicrobial and anticancer region. *J. Am. Chem. Soc.* 128:5776–5785. https://doi.org/10.1021/ja0584875.

43. Wang, G. 2008. Structures of human host defense cathelicidin LL-37 and its smallest antimicrobial peptide KR-12 in lipid micelles. *J. Biol. Chem.* 283:32637–32643. https://doi.org/10.1074/jbc.M805533200.

44. Engelberg, Y., and M. Landau. 2020. The Human LL-37(17-29) antimicrobial peptide reveals a functional supramolecular structure. *Nat. Commun.* 11:3894. https://doi.org/10.1038/s41467-020-17736-x.

45. Geoghegan, J. A., A. D. Irvine, and T. J. Foster. 2018. Staphylococcus aureus and atopic dermatitis: a complex and evolving relationship. *Trends Microbiol.* 26:484–497. https://doi.org/10.1016/j.tim.2017.11.008.

46. Chng, K. R., A. S. Tay, …, N. Nagarajan. 2016. Whole metagenome profiling reveals skin microbiome-dependent susceptibility to atopic dermatitis flare. *Nat. Microbiol.* 1:16106. https://doi.org/10.1038/nmicrobiol.2016.106.

47. Freire, R. V. M., Y. Pillco-Valencia, …, S. Salentinig. 2021. Antimicrobial peptide induced colloidal transformations in bacteria-mimetic vesicles: combining in silico tools and experimental methods. *J. Colloid Interface Sci.* 596:352–363. https://doi.org/10.1016/j.jcis.2021.03.060. https://www.sciencedirect.com/science/article/pii/S0021979721003532.

48. Janosi, L., and A. A. Gorfe. 2010. Simulating POPC and POPC/POPG bilayers: conserved packing and altered surface reactivity. *J. Chem. Theor. Comput.* 6:3267–3273. https://doi.org/10.1021/ct100381g.

49. Sohlenkamp, C., and O. Geiger. 2015. Bacterial membrane lipids: diversity in structures and pathways. *FEMS Microbiol. Rev.* 40:133–159. https://doi.org/10.1093/femsre/fuv008.

50. Rocha-Roa, C., J. D. Orjuela, …, C. Aponte-Santamaría. 2021. Cardiolipin prevents pore formation in phosphatidylglycerol bacterial membrane models. *FEBS Lett.* https://doi.org/10.1002/1873-3468.14206.

51. Blumer, M., S. Harris, …, W. F. D. Bennett. 2020. Simulations of asymmetric membranes illustrate cooperative leaflet coupling and lipid adaptability. *Front. Cell Dev. Biol.* 8. https://doi.org/10.3389/fcell.2020.00575.

52. Sancho-Vaello, E., D. Gil-Carton, …, K. Zeth. 2020. The structure of the antimicrobial human cathelicidin LL-37 shows oligomerization and channel formation in the presence of membrane mimics. *Sci. Rep.* 10:17356. https://doi.org/10.1038/s41598-020-74401-5.

53. Majewska, M., V. Zamlynny, …, P. Pieta. 2021. Interaction of LL-37 human cathelicidin peptide with a model microbial-like lipid membrane. *Bioelectrochemistry.* 141:107842. https://doi.org/10.1016/j.bioelechem.2021.107842. https://www.sciencedirect.com/science/article/pii/S1567539421001055.

Biophysical Journal, Volume *121*

Supplemental information

Small molecules enhance the potency of natural antimicrobial peptides

Valeria Losasso, Khushbu Agarwal, Morris Waskar, Amitabha Majumdar, Jason Crain, Martyn Winn, and Michael Hoptroff

Supporting information

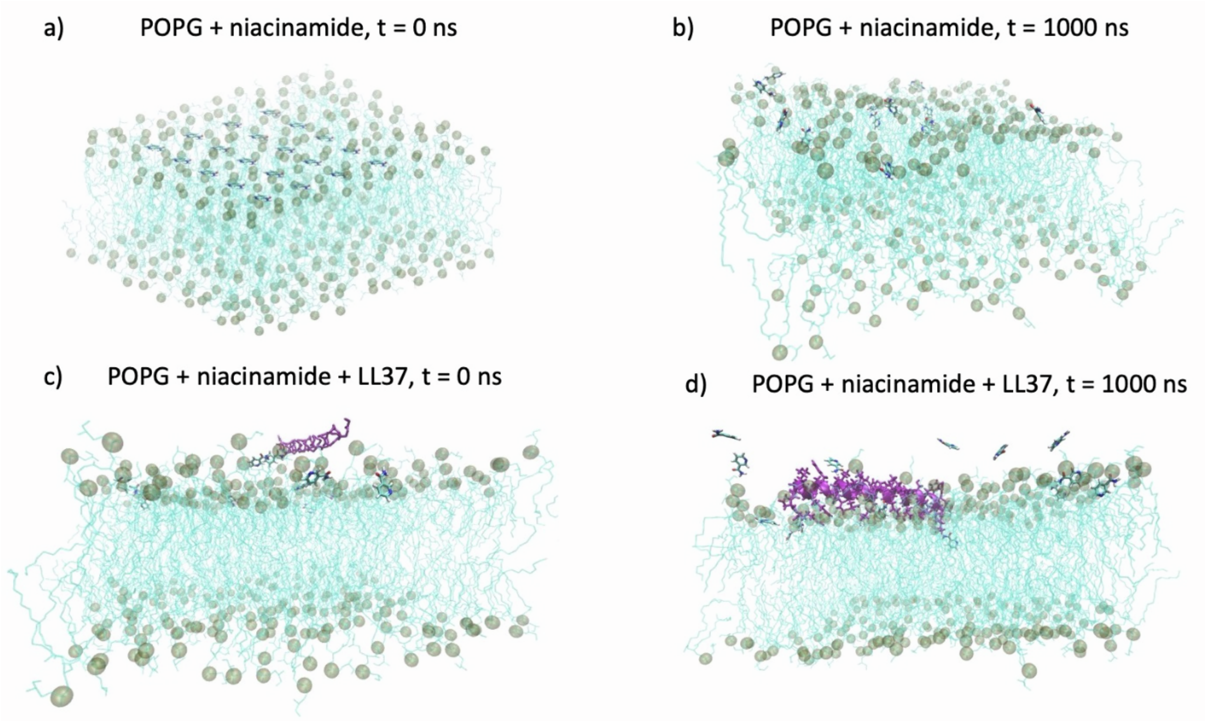

Fig. S1. a) Simulation setup for a membrane system (POPG as an example) with 20 additives in a grid (niacinamide as an example). b) Snapshot of the first POPG + niacinamide simulation at t = 1000 ns. c) Addition of LL-37 peptide onto the system. d) Snapshot of the first POPG + LL-37 + niacinamide simulation at t = 1000 ns.

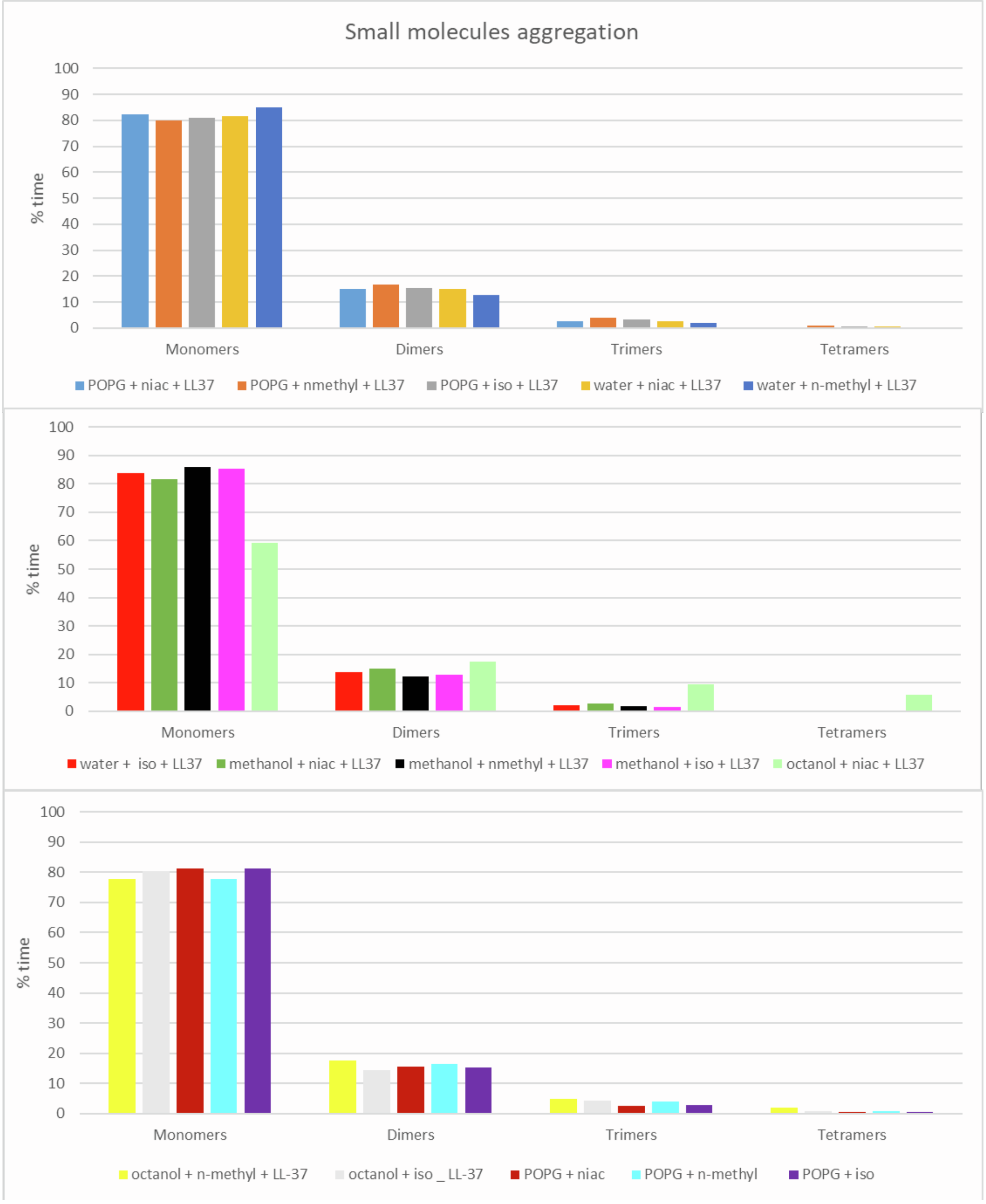

Fig. S2. Percentage over simulation time of aggregates in different sizes for the three additives in membrane, water, methanol and octanol. In every group, at least 1 molecule is located < 5 Å away from any of the others.

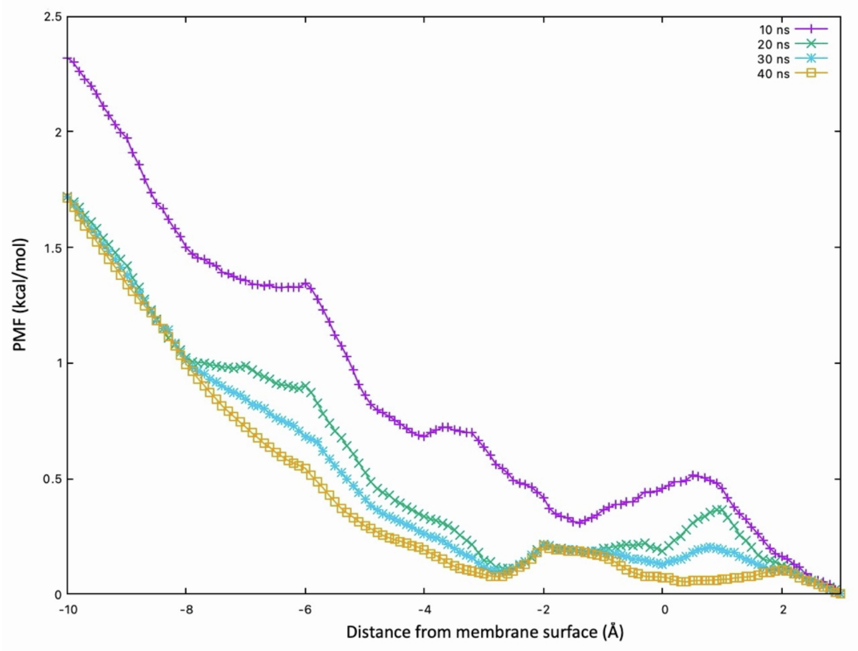

Fig. S3. PMF profile calculated after 10, 20, 30 and 40 ns for POPG + niacinamide.

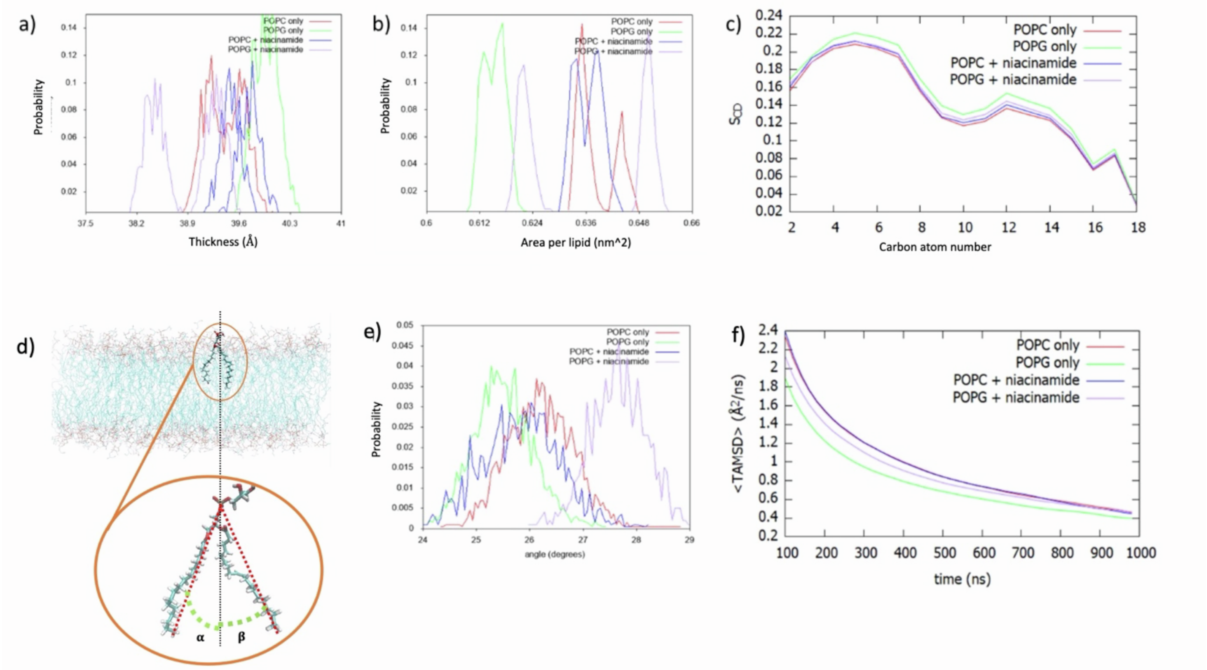

Fig. S4. Analysis of unbiased simulations for niacinamide in complex with POPC or POPG membranes. a) Distribution of thickness values; b) Distribution of area per lipid values; c) S_CD order parameter; d) Definition of tilt angle as the average of α and β angles, formed between the membrane normal at t = 0 and the line connecting the lipid phosphate to the terminal carbon atom of each tail; e) Distribution of lipid tilt angles; f) Ensemble averaged time-averaged mean square displacement (TAMSD).

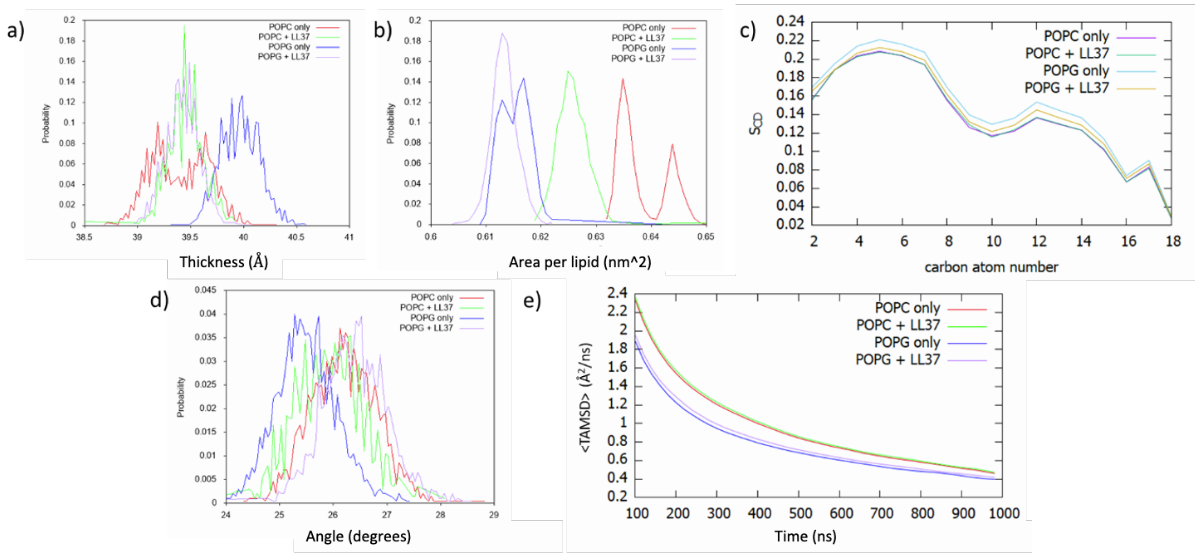

Fig. S5. Analysis of LL-37 in complex with POPC and POPG membranes compared with membrane-only systems. a) Distribution of thickness values; b) Distribution of area per lipid values; c) S_CD order parameter; d) Distribution of lipid tilt angles; e) Ensemble averaged time-averaged mean square displacement (TAMSD).

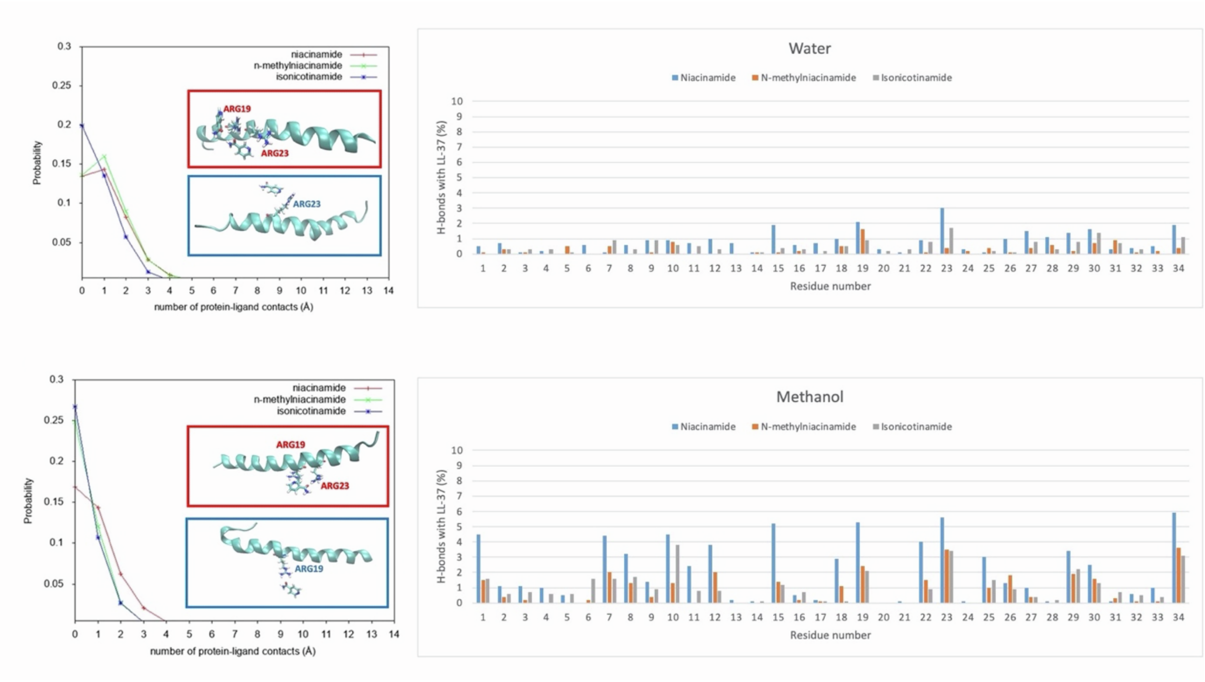

Fig. S6. Distribution of contacts for niacinamide and its derivatives with LL-37 in membrane (top) and octanol (bottom). Representative snapshots for niacinamide and isonicotinamide binding are shown in red and blue boxes, respectively.

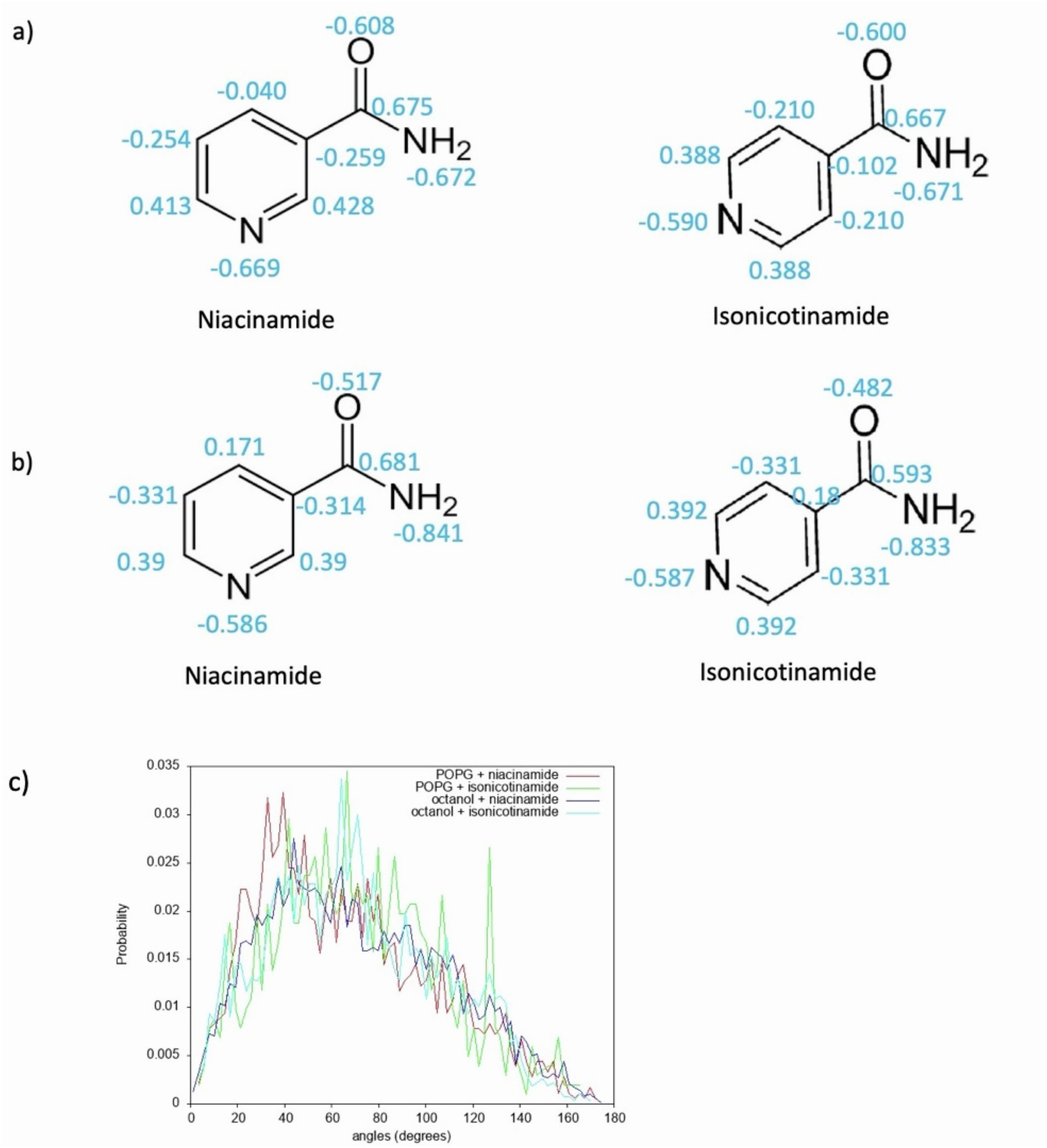

Fig. S7. a) Different charge distribution between niacinamide and isonicotinamide as calculated by Antechamber. b) Partial charges on the two molecules as calculated by restrained electrostatic potential (ESP)-based charge fitting. c) Distribution of angles between LL-37 main axis and niacinamide/isonicotinamide main axis when the two molecules are less than 5 Å apart.

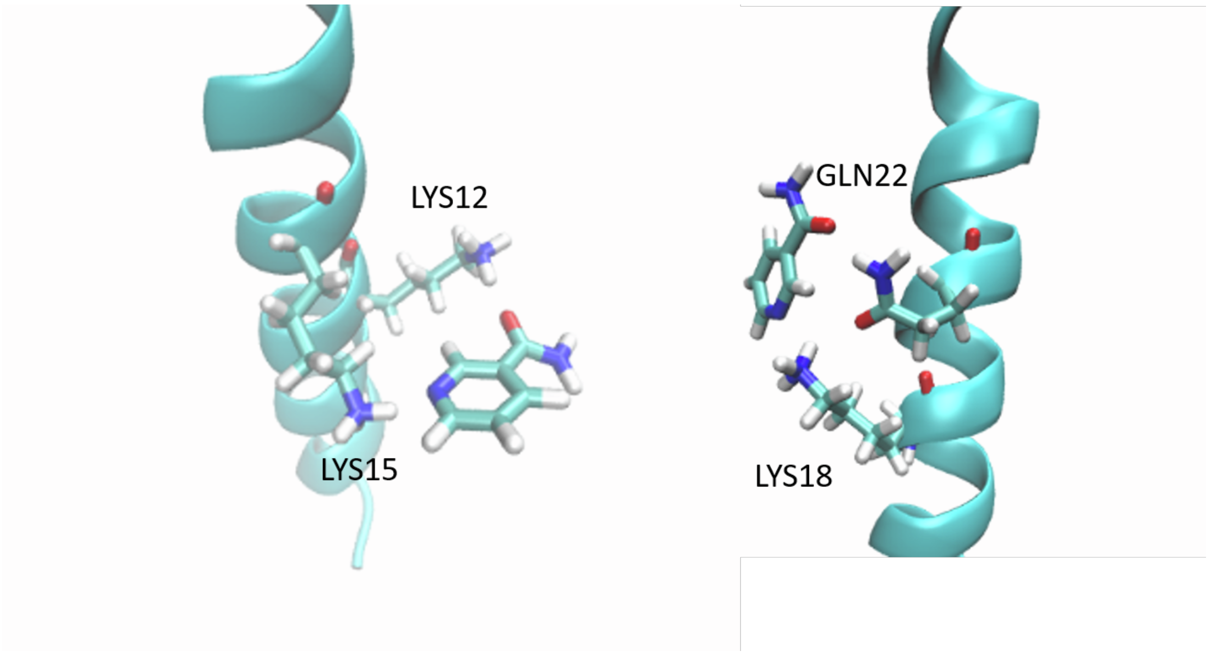

Fig. S8. Simultaneous hydrogen bonds between niacinamide and LL-37 residue pairs.

Table S1. Antimicrobial activity of niacinamide and analogues with LL37 on *S.aureus*

SET 1	Log counts (cfu/mL)		
	R1	R2	Average
Culture Control	1130000	1220000	1175000
3% NIA	1120000	1590000	1355000
3% MeNAM	1270000	1440000	1355000
3% ISON	1350000	1090000	1220000
LL37 (2 µg/mL)	80800	67200	74000
3% NIA + LL37(2 µg/mL)	6100	5100	5600
3% MeNAM + LL37(2 µg/mL)	14800	11300	13050
3% ISON + LL37(2 µg/mL)	57600	41200	49400

SET 2	Log counts (cfu/mL)		
	R1	R2	Average
Culture Control	2480000	2320000	2400000
3% NIA	2400000	2480000	2440000
3% MeNAM	2560000	2480000	2520000
3% ISON	2160000	2400000	2280000
LL37 (2 µg/mL)	22400	19600	21000
3% NIA + LL37(2 µg/mL)	1300	1000	1150
3% MeNAM + LL37(2 µg/mL)	2300	1500	1900
3% ISON + LL37(2 µg/mL)	4500	5300	4900

Key: NIA= Niacinamide, MeNAM= N-methylnicotinamide, ISON= Isonicotinamide,
LL37=Cathelicidin Antimicrobial peptide (AMP)
All % values as w/v
R1 and R2 are technical replicates

Table S2. Hydrogen bonds between niacinamide, n-methylnicotinamide and nicotinamide and LL-37 in four different environments (POPG membrane, water, methanol, octanol).

	Ring N	Amide N	Amide O
POPG + niacinamide	Leu2 0.3%<br>Phe5 0.2%<br>Ser9 0.6%<br>Arg7 0.4%<br>Lys8 0.2%<br>Lys10 0.3%<br>Lys15 0.2%<br>Lys18 2.2%<br>Lys25 0.6%<br>Arg23 0.6%<br>Gln22 1%<br>Arg29 0.4%<br>Asn30 0.4%<br>Arg34 0.3%	Leu1 0.4%<br>Leu2 0.7%<br>Gly3 0.2%<br>Asp4 0.4%<br>Phe6 0.2%<br>Arg7 0.5%<br>Ser9 0.4%<br>Lys10 0.2%<br>Glu11 0.6%<br>Lys12 0.1%<br>Ile13 0.2%<br>Gly14 0.2%<br>Lys15 0.3%<br>Glu16 0.3%<br>Lys18 0.3%<br>Arg19 0.6%<br>Ile20 0.3%<br>Val21 0.1%<br>Gln22 2.6%<br>Arg23 0.7%<br>Ile24 0.1%<br>Lys25 0.3%<br>Asp26 1%<br>Phe27 0.2%<br>Leu28 0.2%<br>Arg29 0.4%<br>Asn30 0.6%<br>Leu31 0.3%<br>Val32 0.2%<br>Pro33 0.7%<br>Arg34 0.4%	Leu1 0.9%<br>Leu2 0.3%<br>Gly3 0.9%<br>Asp4 0.1%<br>Phe6 0.1%<br>Arg7 0.4%<br>Lys8 0.4%<br>Ser9 0.1%<br>Lys10 1%<br>Glu11 0.3%<br>Lys12 0.3%<br>Lys15 0.5%<br>Glu16 0.1%<br>Lys18 0.8%<br>Arg19 1%<br>Val21 0.2%<br>Gln22 2.1%<br>Arg23 0.5%<br>Ile24 0.1%<br>Lys25 0.9%<br>Asp26 0.2%<br>Phe27 0.1%<br>Arg29 0.6%<br>Asn30 0.7%<br>Arg34 0.6%
POPG + n-methylnicotinamide	Leu1 0.1%<br>Leu2 0.1%<br>Gly3 0.1%<br>Arg7 0.1%<br>Lys8 0.2%<br>Ser9 0.5%<br>Lys10 0.3%<br>Lys12 0.3%<br>Lys18 0.1%<br>Arg19 1.2%<br>Arg23 0.5%<br>Ile24 0.1%	Leu1 0.3%<br>Leu2 0.2%<br>Phe5 0.4%<br>Lys8 0.1%<br>Ser9 1.2%<br>Lys10 0.2%<br>Glu11 0.2%<br>Lys12 1%<br>Ile13 1%<br>Glu16 0.9%<br>Phe17 1%<br>Gln22 0.1%	Leu1 0.2%<br>Leu2 0.3%<br>Gly3 0.2%<br>Phe5 0.1%<br>Phe6 0.1%<br>Lys8 0.1%<br>Ser9 1.4%<br>Lys10 0.9%<br>Lys12 0.9%<br>Ile13 0.3%<br>Lys15 0.1%<br>Lys18 0.3%

	Lys25 0.3%<br>Arg29 0.1%<br>Asn30 0.9%<br>Arg34 0.4%	Arg23 1.1%<br>Asp26 0.6%<br>Phe27 0.3%<br>Arg29 0.2%<br>Asn30 1.9%<br>Leu31 0.7%<br>Pro33 0.2%<br>Arg34 0.1%	Arg19 1%<br>Ile20 0.3%<br>Val21 0.1%<br>Gln22 0.5%<br>Arg23 2.6%<br>Ile24 0.3%<br>Lys25 3.8%<br>Phe27 0.2%<br>Leu28 0.4%<br>Arg29 1.4%<br>Asn30 1.1%<br>Leu31 0.2%<br>Val32 0.1%<br>Arg34 2%
POPG +<br>isonicotinamide	Arg7 0.1%<br>Ser9 0.2%<br>Lys10 0.1%<br>Lys12 0.1%<br>Lys15 0.1%<br>Arg19 0.2%<br>Gln22 0.2%<br>Arg23 0.1%<br>Lys25 0.6%<br>Arg29 0.2%<br>Asn30 0.3%<br>Arg34 1.3%	Leu1 0.1%<br>Gly3 0.1%<br>Phe5 0.1%<br>Phe6 0.1%<br>Arg7 0.3%<br>Ser9 0.4%<br>Lys10 0.2%<br>Glu11 0.2%<br>Lys12 0.5%<br>Ile13 0.1%<br>Lys15 0.1%<br>Glu16 0.5%<br>Phe17 0.1%<br>Lys18 1.1%<br>Arg19 0.1%<br>Ile20 0.1%<br>Gln22 0.7%<br>Arg23 1.3%<br>Ile24 0.1%<br>Lys25 0.9%<br>Asp26 0.8%<br>Phe27 0.8%<br>Leu28 0.4%<br>Arg29 0.6%<br>Asn30 0.9%<br>Leu31 0.7%<br>Leu32 0.6%<br>Pro33 0.2%<br>Arg34 1.4%	Gly3 0.3%<br>Phe6 0.2%<br>Arg7 0.3%<br>Lys8 0.1%<br>Ser9 0.1%<br>Lys10 0.5%<br>Lys12 0.5%<br>Phe17 0.3%<br>Lys18 1%<br>Gly14 0.1%<br>Arg19 0.1%<br>Ile20 0.2%<br>Gln22 0.1%<br>Arg23 1.4%<br>Lys25 1.3%<br>Asp26 0.1%<br>Arg29 0.2%<br>Arg30 0.9%<br>Pro33 0.1%<br>Arg34 1.4%
niacinamide + water	Leu1 0.1%<br>Leu2 0.1%<br>Gly3 0.1%<br>Phe5 0.1%<br>Arg7 0.1%<br>Lys8 0.3%<br>Ser9 0.2%<br>Lys10 0.1%	Leu1 0.2 %<br>Leu2 0.3%<br>Asp4 0.2%<br>Phe5 0.5%<br>Phe6 0.6%<br>Ser9 0.2%<br>Lys10 0.4%<br>Glu11 0.7%	Leu1 0.2%<br>Leu2 0.3%<br>Phe6 0.2%<br>Arg7 0.9%<br>Lys8 0.3%<br>Ser9 0.5%<br>Lys10 0.4%<br>Lys12 0.6%

	Lys12 0.3%<br>Lys15 0.1%<br>Phe17 0.2%<br>Lys18 0.2%<br>Arg19 0.3%<br>Ile20 0.1%<br>Gln22 0.2%<br>Arg23 0.5%<br>Ile24 0.1%<br>Lys25 0.1%<br>Leu28 0.2%<br>Arg29 0.3%<br>Asn30 0.2%<br>Leu31 0.1%<br>Val32 0.2%<br>Arg34 0.4%	Lys12 0.1%<br>Ile13 0.5%<br>Gly14 0.1%<br>Lys15 0.2%<br>Glu16 0.6%<br>Phe17 0.5%<br>Arg19 0.2%<br>Ile20 0.2%<br>Gln22 0.5%<br>Arg23 0.2%<br>Ile24 0.1%<br>Asp26 1%<br>Phe27 1.3%<br>Leu28 0.8%<br>Arg29 0.4%<br>Asn30 0.7%<br>Leu31 0.3%<br>Val32 0.4%<br>Pro33 0.2%<br>Arg34 0.5%	Ile13 0.2%<br>Lys15 0.4%<br>Lys18 0.8%<br>Arg19 1.6%<br>Ile20 0.1%<br>Ile24 0.1%<br>Lys15 1.2%<br>Val21 0.1%<br>Gln22 0.2%<br>Arg23 2.3%<br>Phe27 0.2%<br>Leu28 0.1%<br>Arg29 0.7%<br>Asn30 0.7%<br>Leu31 0.3%<br>Val32 0.4%<br>Pro33 0.3%<br>Arg34 1%
N-methylnicotinamide + water	Lys10 0.3%<br>Arg19 0.1%<br>Gln22 0.1%<br>Lys25 0.3%<br>Phe27 0.1%<br>Asn30 0.1%	Leu1 0.1%<br>Leu2 0.3%<br>Gly3 0.1%<br>Phe6 0.1%<br>Ser9 0.1%<br>Lys10 0.7%<br>Glu16 0.2%<br>Arg19 1.3%<br>Arg23 0.2%<br>Lys25 0.1%<br>Asp26 0.1%<br>Phe27 0.3%<br>Leu28 0.6%<br>Arg29 0.1%<br>Asn30 0.6%<br>Pro33 0.1%<br>Arg34 0.3%	Arg7 0.5%<br>Lys10 0.1%<br>Gly14 0.1%<br>Lys15 0.1%<br>Lys18 0.5%<br>Arg19 0.2%<br>Arg23 0.2%<br>Ile24 0.2%<br>Arg29 0.1%<br>Leu31 0.1%<br>Arg34 0.1%
Isonicotinamide + water	Leu1 0.1%<br>Leu2 0.1%<br>Gly3 0.1%<br>Ser9 0.2%<br>Lys10 0.1%<br>Glu11 0.1%<br>Lys15 0.1%<br>Phe17 0.1%<br>Arg19 0.1%<br>Gln22 0.2%<br>Lys25 0.4%<br>Leu28 0.1%<br>Asn30 0.3%<br>Arg23 0.3%	Leu1 0.2%<br>Leu2 0.2%<br>Asp4 0.3%<br>Phe5 0.3%<br>Phe6 0.7%<br>Arg7 0.1%<br>Lys8 0.1%<br>Ser9 0.2%<br>Lys10 0.2%<br>Glu11 0.2%<br>Ile13 0.2%<br>Glu16 0.3%<br>Phe17 0.1%<br>Lys18 0.2%	Leu1 0.2%<br>Gly3 0.2%<br>Phe5 0.1%<br>Phe6 0.1%<br>Arg7 0.8%<br>Lys8 0.2%<br>Ser9 0.5%<br>Lys10 0.3%<br>Glu11 0.2%<br>Lys12 0.3%<br>Ile13 0.2%<br>Gly14 0.1%<br>Lys15 0.3%<br>Lys18 0.3%

	Arg34 0.4%	Ile20 0.1%<br>Gln22 0.1%<br>Arg23 0.4%<br>Ile24 0.1%<br>Lys25 0.1%<br>Asp26 0.1%<br>Phe27 0.8%<br>Leu28 0.1%<br>Asn30 0.7%<br>Leu31 0.3%<br>Val32 0.4%<br>Arg34 0.1%	Arg19 0.8%<br>Ile20 0.1%<br>Val21 0.3%<br>Gln22 0.5%<br>Arg23 1.3%<br>Lys25 0.5%<br>Leu28 0.1%<br>Arg29 0.8%<br>Asn30 0.4%<br>Val32 0.1%<br>Arg34 0.6%
Niacinamide + methanol	Leu1 0.1%<br>Leu2 0.1%<br>Gly3 0.1%<br>Phe5 0.2%<br>Arg7 0.3%<br>Ser9 0.6%<br>Lys10 0.3%<br>Lys12 0.5%<br>Lys15 0.5%<br>Lys18 0.4%<br>Arg19 0.4%<br>Gln22 0.3%<br>Arg23 0.5%<br>Lys25 0.3%<br>Arg29 0.1%<br>Asn30 0.1%<br>Arg34 0.2%	Leu1 0.1%<br>Leu2 0.1%<br>Gly3 0.1%<br>Asp4 0.8%<br>Phe5 0.2%<br>Lys8 0.4%<br>Ser9 0.2%<br>Lys10 0.5%<br>Glu11 2.2%<br>Ile13 0.1%<br>Gly14 0.1%<br>Lys15 0.3%<br>Glu16 0.5%<br>Phe17 0.2%<br>Lys18 0.1%<br>Arg19 0.2%<br>Val21 0.1%<br>Gln 22 2.5%<br>Arg23 0.4%<br>Asp26 1.3%<br>Phe27 0.9%<br>Leu28 0.1%<br>Arg29 0.4%<br>Asn30 1.1%<br>Leu31 0.1%<br>Val32 0.5%<br>Pro33 1%<br>Arg34 1.2%	Leu1 4.3%<br>Leu2 0.9%<br>Gly3 0.9%<br>Asp4 0.2%<br>Phe5 0.1%<br>Arg7 4.1%<br>Lys8 2.8%<br>Ser9 0.6%<br>Lys10 3.7%<br>Glu11 0.2%<br>Lys12 3.3%<br>Ile13 0.1%<br>Lys15 4.4%<br>Lys18 2.4%<br>Arg19 4.7%<br>Gln22 1.2%<br>Arg23 4.7%<br>Ile24 0.1%<br>Lys25 2.7%<br>Phe27 0.1%<br>Arg29 2.9%<br>Asn30 1.3%<br>Val32 0.1%<br>Arg34 4.5%
N-methylnicotinamide + methanol	Leu1 0.4%<br>Leu2 0.1%<br>Arg7 0.5%<br>Lys8 0.2%<br>Ser9 0.3%<br>Lys10 0.4%<br>Lys12 0.3%<br>Lys15 0.2%<br>Phe17 0.1%<br>Lys18 0.3%<br>Arg19 0.4%<br>Gln22 0.4%	Asp4 0.3%<br>Glu16 0.2%<br>Gln22 0.3%<br>Lys25 0.1%<br>Asp26 1.8%<br>Phe27 0.4%<br>Arg29 0.2%<br>Asn30 0.6%<br>Leu31 0.3%<br>Val32 0.1%<br>Arg34 0.8%	Leu1 1.1%<br>Leu2 0.3%<br>Gly3 0.2%<br>Phe6 0.2%<br>Arg7 2%<br>Lys8 1.1%<br>Ser9 0.1%<br>Lys10 0.9%<br>Lys12 1.7%<br>Lys15 1.2%<br>Lys18 0.8%<br>Arg19 2%

	Arg23 0.7%<br>Lys25 0.1%<br>Arg29 0.3%<br>Asn30 0.5%<br>Arg34 0.9%		Gln22 0.8%<br>Arg23 2.8%<br>Lys25 0.8%<br>Arg29 1.4%<br>Asn30 0.5%<br>Pro33 0.1%<br>Arg34 1.9%
Isonicotinamide + methanol	Gly3 0.1%<br>Phe5 0.1%<br>Arg7 0.1%<br>Lys8 0.1%<br>Ser9 0.2%<br>Lys10 0.3%<br>Lys15 0.2%<br>Lys18 0.1%<br>Gln22 0.3%<br>Arg23 0.2%<br>Lys25 0.2%<br>Arg29 0.3%<br>Asn30 0.3%<br>Arg34 0.4%	Leu1 0.2%<br>Leu2 0.1%<br>Gly3 0.2%<br>Asp4 0.6%<br>Arg7 0.1%<br>Ser9 0.4%<br>Lys10 0.4%<br>Glu11 0.8%<br>Lys12 0.1%<br>Gly14 0.1%<br>Lys15 0.2%<br>Glu16 0.7%<br>Phe17 0.1%<br>Gln22 0.2%<br>Arg23 0.1%<br>Lys25 0.2%<br>Asp26 0.9%<br>Phe27 0.2%<br>Leu28 0.1%<br>Arg29 0.5%<br>Asn30 0.3%<br>Leu31 0.7%<br>Val32 0.5%<br>Pro33 0.3%<br>Arg34 0.9%	Leu1 1.4%<br>Leu2 0.5%<br>Gly3 0.4%<br>Phe5 0.5%<br>Arg7 1.4%<br>Lys8 1.6%<br>Ser9 0.3%<br>Lys10 3.1%<br>Lys12 0.7%<br>Lys15 0.8%<br>Lys18 1.5%<br>Arg19 2.1%<br>Gln22 0.4%<br>Arg23 3.1%<br>Lys25 1.1%<br>Phe27 0.2%<br>Leu28 0.1%<br>Arg29 1.4%<br>Asn30 0.7%<br>Pro33 0.1%<br>Arg34 1.8%
Niacinamide + octanol	Leu1 0.2%<br>Leu2 0.4%<br>Phe5 0.1%<br>Arg7 1.3%<br>Lys8 9.3%<br>Ser9 6.3%<br>Lys10 1%<br>Lys12 8.6%<br>Ile13 0.1%<br>Lys15 31.3%<br>Phe17 0.1%<br>Lys18 53.6%<br>Arg19 2.8%<br>Ile20 0.6%<br>Val21 0.6%<br>Gln22 21.7%<br>Arg23 0.9%<br>Ile24 0.4%<br>Lys25 2.1%	Leu1 1.6%<br>Asp4 5.5%<br>Phe5 1.1%<br>Lys8 3.4%<br>Ser9 0.5%<br>Lys10 0.1%<br>Glu11 16.8%<br>Lys12 0.2%<br>Ile13 0.2%<br>Lys15 0.1%<br>Glu16 12.7%<br>Phe17 0.7%<br>Arg19 2.1%<br>Ile20 0.4%<br>Val21 0.1%<br>Gln22 49%<br>Ile24 0.2%<br>Lys25 0.5%<br>Asp26 5.1%	Leu1 22.9%<br>Leu2 9.6%<br>Gly3 3.6%<br>Asp4 1%<br>Phe5 0.2%<br>Phe6 0.4%<br>Arg7 25.6%<br>Lys8 41.5%<br>Ser9 4.8%<br>Lys10 41%<br>Lys12 87.8%<br>Ile13 0.2%<br>Lys15 51.9%<br>Glu16 0.2%<br>Lys18 53.1%<br>Arg19 74.6%<br>Ile20 0.7%<br>Val21 0.4%<br>Gln22 16%

	Arg29 1.9%<br>Asn30 0.7%<br>Leu31 0.1%<br>Val32 0.7%<br>Arg34 0.3%	Phe27 1.7%<br>Leu28 0.6%<br>Arg29 10.1%<br>Asn30 13.1%<br>Leu31 0.1%<br>Val32 33.3%<br>Pro33 2.5%<br>Arg34 7.4%	Arg23 47.1%<br>Lys25 54.1%<br>Phe27 1%<br>Leu28 0.3%<br>Arg29 31.6%<br>Asn30 13.3%<br>Leu31 0.6%<br>Val32 1.70%<br>Arg34 10.4%
N-methylnicotinamide + octanol	Leu1 4.9%<br>Leu2 2.3%<br>Gly3 3.1%<br>Asp4 0.1%<br>Phe5 0.2%<br>Phe6 0.1%<br>Arg7 11.9%<br>Lys8 2.2%<br>Ser9 22.5%<br>Lys10 1.6%<br>Lys12 3.9%<br>Lys15 11.50%<br>Lys18 2.2%<br>Arg19 1.2%<br>Gln22 6.2%<br>Arg23 1.3%<br>Lys25 2.7%<br>Asn30 0.4%<br>Leu31 0.3%<br>Val32 1.7%<br>Arg34 0.4%	Gln22 3%<br>Asp26 0.1%<br>Leu28 2.5%<br>Arg29 1.9%<br>Asn30 0.7%<br>Leu31 8.8%<br>Val32 0.4%<br>Pro33 0.1%<br>Arg34 3.2%	Leu1 15.8%<br>Leu2 9.9%<br>Gly3 12.8%<br>Phe5 0.1%<br>Phe6 0.3%<br>Arg7 19.1%<br>Lys8 71.3%<br>Ser9 16.6%<br>Lys10 11%<br>Lys12 97.1%<br>Ile13 0.1%<br>Lys15 18.4%<br>Lys18 15.5%<br>Arg19 25.9%<br>Val21 0.1%<br>Gln22 4.5%<br>Arg23 4.7%<br>Lys25 30.8%<br>Phe27 0.1%<br>Leu28 0.2%<br>Asn30 2%<br>Leu31 0.3%<br>Val32 2.8%<br>Pro33 0.3%<br>Arg34 9.1%
Isonicotinamide + octanol	Leu1 0.2%<br>Phe5 0.1%<br>Phe6 0.2%<br>Arg7 1.2%<br>Ser9 1.1%<br>Lys10 0.7%<br>Lys12 0.4%<br>Lys18 0.4%<br>Arg19 0.5%<br>Gln22 4.5%<br>Arg23 0.4%<br>Lys25 1.2%<br>Arg29 0.1%<br>Asn30 1.3%<br>Pro33 0.1%<br>Arg34 0.5%	Leu1 0.6%<br>Asp4 1.6%<br>Phe5 0.1%<br>Phe6 0.2%<br>Ser9 1%<br>Glu11 0.8%<br>Glu16 1%<br>Arg19 0.3%<br>Ile20 0.2%<br>Val21 0.2%<br>Gln22 1.7%<br>Arg23 0.2%<br>Lys25 0.2%<br>Asp26 2.1%<br>Phe27 0.1%<br>Arg29 3.1%	Leu1: 40%<br>Leu2: 2.8%<br>Gly3: 17.2%<br>Asp4: 0.5%<br>Phe5: 0.1%<br>Arg7: 11%<br>Lys8: 11%<br>Ser9: 4.3%<br>Lys10:34.7%<br>Lys12: 12.3%<br>Lys15:13.9%<br>Glu16:0.1%<br>Phe17:0.1%<br>Lys18:21.1%<br>Arg19:30.4%<br>Val21:0.1%

		Asn30 5.2%<br>Leu31 0.4%<br>Val32 2.1%<br>Pro33 4.6%<br>Arg34 25.7%	Gln22:23.6%<br>Arg23:6.3%<br>Lys25:14.9%<br>Asp26:0.3%<br>Phe27:0.1%<br>Arg29:12%<br>Asn30:4.9%<br>Arg34:15.4%
